# Ultrasound-mediated structural and chemical modifications in wheat proteins and their impact on functional and storage properties: a review

**DOI:** 10.1039/d6ra03737b

**Published:** 2026-10-19

**Authors:** Kanu Garg, Shubhra Sinha, Kamaljit Kaur, Ovais Shafiq Qadri

**Affiliations:** a Department of Biotechnology, Thapar Institute of Engineering and Technology Patiala Punjab 147004 India osqadri@thapar.edu; b TIET-TAU-PAU Centre of Excellence for Food Security, Thapar Institute of Engineering and Technology Patiala Punjab 147004 India; c Department of Food Science and Technology, Punjab Agricultural University Ludhiana Punjab 141004 India

## Abstract

Wheat is one of the most widely consumed cereal grains across the world because of its versatile adaptability and applications. Nearly 25% of the global cereal production is contributed by wheat. It is a rich source of carbohydrates, proteins, dietary fibres, and vitamins. While wheat is generally a stable food with low moisture content, multiple interventions have been researched on the enhancement of the functional, morphological, and physicochemical properties of protein, starch, and dietary fibres of wheat. In addition to modification of the properties, an increase in shelf life of the product has also been explored through different processing techniques. Among the novel methods, ultrasonication is a non-thermal food processing technology that enhances shelf life and quality of food. Conventional thermal processing techniques help in improving shelf life but can destroy heat-sensitive nutrients. Ultrasonication provides a practical solution by simultaneously improving shelf life and quality and preserving thermally sensitive nutrients. Ultrasonication has been proven efficient in attaining sustainability, enhancing efficiency, and ensuring precision in food production and quality control. Ultrasonication has been used extensively on wheat and wheat-based products. Numerous studies are focused on using ultrasound to modify the properties of the dough. Other studies focus on the effect of ultrasound on the structure of the wheat grain components like starch and proteins. This review provides a comprehensive overview of studies that involve wheat and ultrasonication. It reviews the effects of ultrasonication on the composition and functionality of the grain. The mechanism by which ultrasonication contributes to preservation by enzyme microbial inactivation has also been reviewed. Various studies on ultrasonication of wheat grains, dough, or other products have been summarized, and their results, which have been validated by various analytical techniques like SEM, FT-IR technology, and texture analysis, are discussed.

## Abstract 2

Schematic representation of ultrasound treatment, characterization, structural modifications, functional properties, and applications of wheat and wheat-based materials

## Graphical abstract

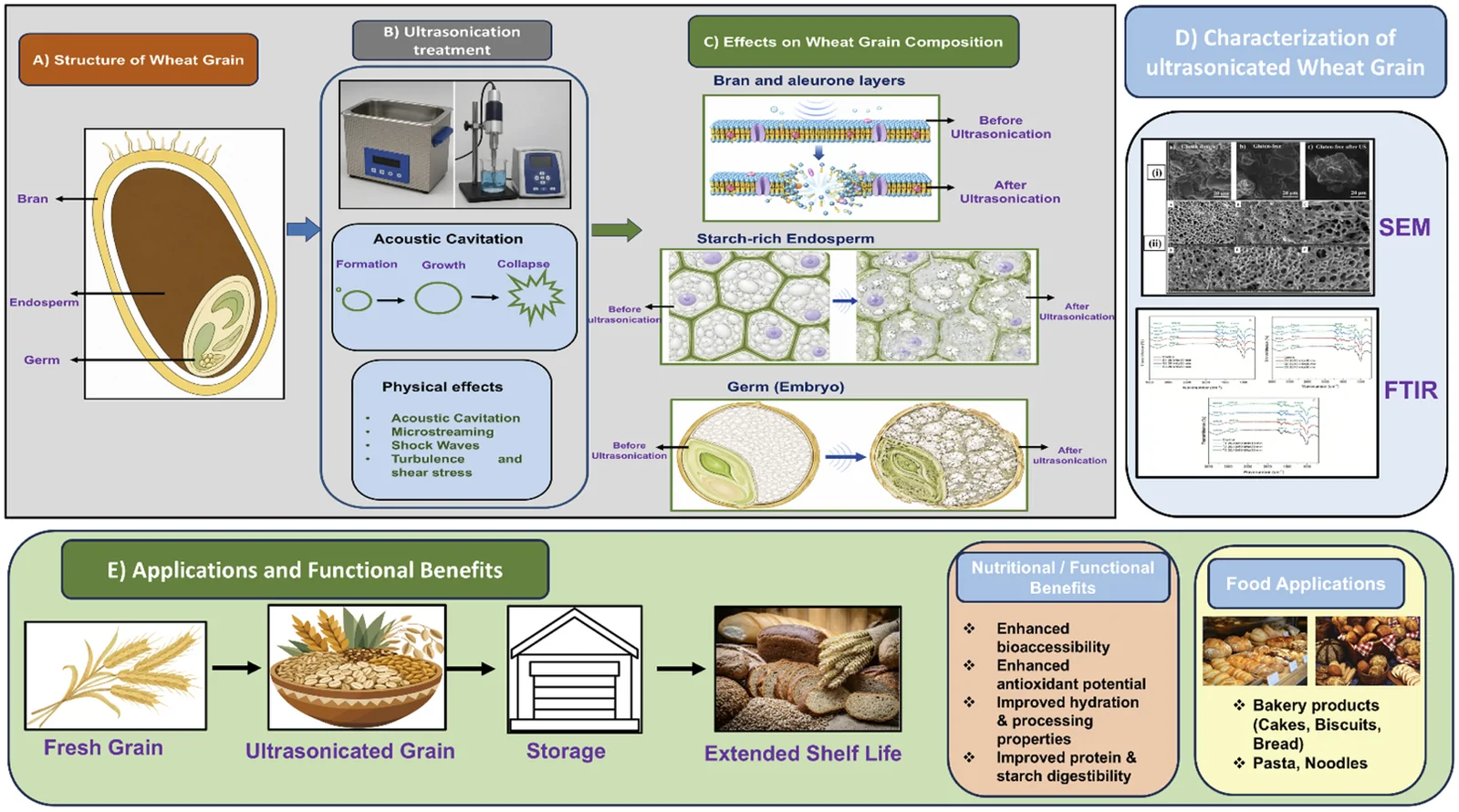

## 1. Introduction

Wheat (*Triticum aestivum* L.) is one of the most widely cultivated and consumed cereal grains globally, owing to its agronomic adaptability, ease of storage, and versatility in food applications.^1^ Annual global wheat production ranges from 700 to 770 million tons, underscoring its significance as a major contributor to food and nutritional security.^2^ Wheat accounts for nearly 25% of total cereal production worldwide and is an important source of carbohydrates, proteins, dietary fiber, essential amino acids, and vitamins, contributing approximately 20% of daily energy intake and 25% of dietary protein consumption globally.^3^

In the past twenty years, three nations in the Black Sea Region—Russia, Ukraine, and Kazakhstan—have emerged as world leaders in grain production and trade, surpassing the USA and France as the top exporters of wheat. By country, Russia accounted for 35% of the recent growth in global wheat exports, followed by Ukraine at 12% and Kazakhstan at 4%. In contrast, Australia and the United States experienced declining shares in wheat export growth.^4^ However, this rapid growth and processing generate substantial agricultural residues and processing wastes. The European Union, Russia, and the United States were the major wheat exporters in 2020/21. However, China and India were the main wheat producers rather than Russia and the United States. The three major exporters generated up to 32.3 million metric tons of wheat grain residues rich in cellulose.^5^

Wheat consumption has increased faster than that of other cereal crops, resulting in substantial quantities of agricultural residue. Notably, wheat straw contains a high proportion of fibrous tissue (∼67%), which is exceptionally high compared to other cereal biomasses. Because traditional petroleum-based plastics pose severe environmental challenges, sustainable alternatives are urgently needed. In this context, utilizing wheat residue and wheat starch to engineer biocomposite materials offers a highly promising, eco-friendly substitute for synthetic petroleum plastics.^6^

Wheat bran, a by-product of the dry milling of wheat, is primarily utilized in the animal feed industry and has also attracted attention because of its high phytochemical and dietary fiber content, which provides beneficial physiological effects.^7^ Wheat straw is one of the most abundant agricultural residues and is produced in enormous quantities. It can be used for bioethanol production through microbial fermentation. Bioethanol is an important type of alcohol with properties similar to ethanol and is produced when microorganisms ferment sugars, carbohydrates, and starches present in plants such as sugarcane, corn, sweet sorghum, and cellulose-rich biomass.^8^

Botanically, wheat is a self-pollinating allohexaploid species (2*n* = 6*x* = 42, AABBDD), a genetic configuration that enables successful cultivation across diverse temperate agro-climatic conditions.^9^ Among cultivated species, common bread wheat (*Triticum aestivum* L.) constitutes about 95% of total global production, whereas durum wheat (*Triticum durum* L.) contributes roughly 5%. Other wheat species, such as emmer (*Triticum dicoccum*), einkorn (*Triticum monococcum*), and spelt (*Triticum spelta*), are produced on a limited scale, primarily for specialty and traditional food products.^10^

The wheat kernel is typically oval in shape as shown in Fig. 1 and is composed of three main anatomical fractions: the endosperm, which represents approximately 82% of the kernel weight; the bran, accounting for about 15%; and the germ or embryo, comprising nearly 3%. The bran fraction includes multiple layers such as the epicarp, endocarp, testa, nucellar epidermis, and aleurone layer, with the aleurone alone contributing close to 50% of total bran mass. In terms of composition, starch is the predominant constituent of wheat grain (≈70%), followed by proteins (8–20%), while lipids, minerals, vitamins, terpenoids, and phenolic compounds are present in smaller quantities. Notably, bran is particularly rich in dietary fiber and phenolic compounds, which play a critical role in water distribution, dough handling properties, and the sensory quality of wheat-based products.^11–13^

**Fig. 1:**
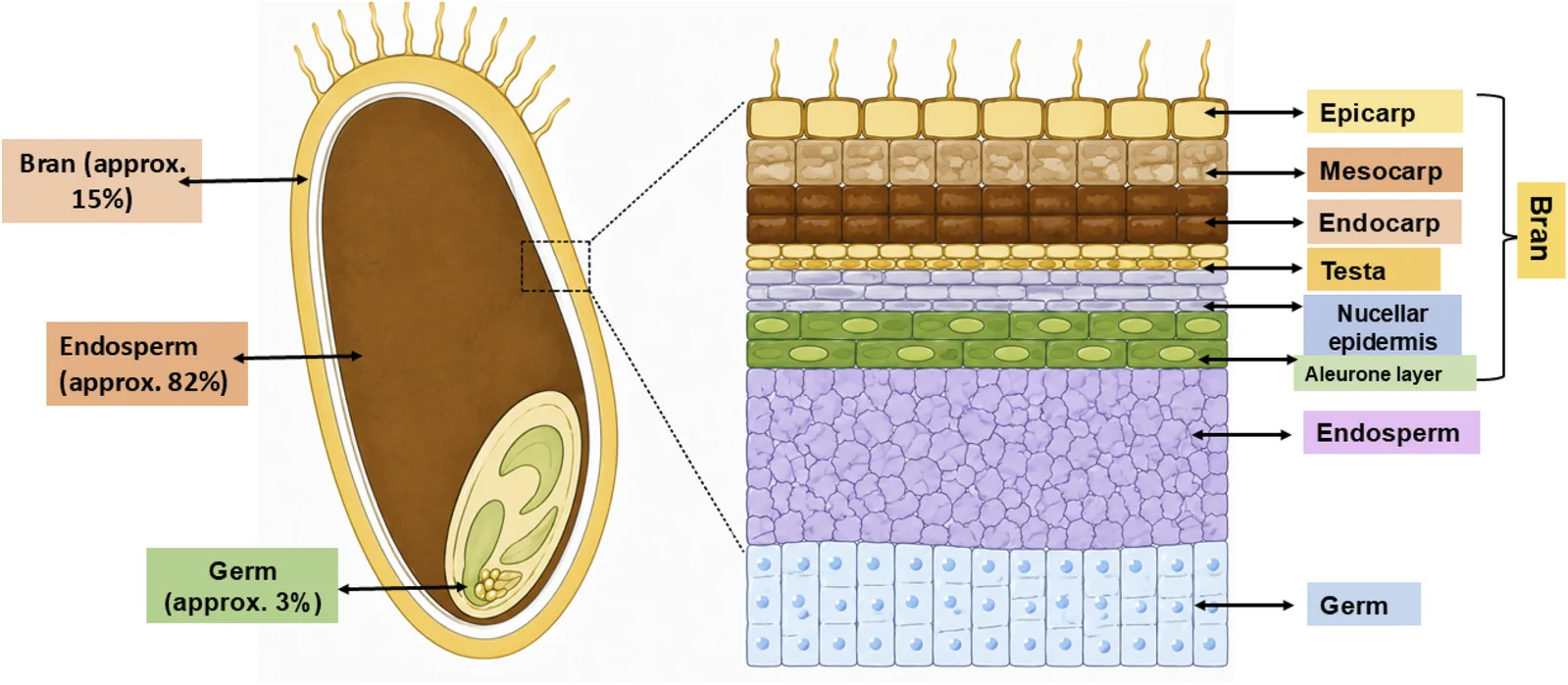
Schematic representation of the anatomical structure and major components of a wheat kernel.

Commercial wheat processing is dominated by refined flour production *via* conventional roller milling, which involves multiple stages of grinding, sieving, and purification. While these processes enhance flour whiteness, texture, and processing performance, they also lead to significant nutrient losses and yield products that are rapidly digested and absorbed. Frequent consumption of refined wheat products has been associated with postprandial hyperglycaemia and an increased risk of chronic metabolic disorders.^14,15^ In contrast, whole wheat flour retains bran and germ fractions, preserving nutritional integrity; however, the presence of endogenous lipids and enzymes in these fractions accelerates oxidative rancidity and enzymatic degradation, thereby limiting shelf life. Consequently, there is a growing need for processing strategies that can retain the nutritional benefits of whole wheat while maintaining desirable functional properties and improving storage stability.

In recent years, non-thermal food processing technologies have gained increasing attention as viable alternatives to conventional thermal treatments, particularly for preserving nutritional and sensory attributes. Among these, ultrasonication has emerged as an environmentally friendly and effective technique with promising applications in cereal processing. Ultrasonic waves are sound waves with frequencies above the audible range (>15–20 kHz), generated using piezoelectric or magnetostrictive transducers that transmit high-energy vibrations to a probe or sonotrode in contact with the food matrix.^16^ The fundamental mechanism of ultrasonication is acoustic cavitation, during which microscopic bubbles form, grow, and collapse rapidly within the medium, generating intense localized shear forces and transient high-energy conditions capable of modifying molecular interactions.

Ultrasound is commonly classified into low-frequency, high-power ultrasound (20–100 kHz), medium-frequency sonochemical ultrasound (100 kHz–2 MHz), and high-frequency ultrasound (5–10 MHz), primarily used for medical and analytical applications.^17^ In food systems, high-power ultrasound (>5 W cm^−2^) is particularly relevant, as it can alter physicochemical properties, inactivate enzymes, and reduce microbial load.^18^ In contrast, low-power ultrasound is a simple, cost-effective, non-invasive, and non-destructive analytical technique used to evaluate the physicochemical and structural properties of food materials, determine their composition, and detect contaminants such as metal fragments and other foreign materials.^19^ In wheat and wheat-based systems, ultrasonication has been reported to enhance the functional and structural properties of starch, proteins, and dietary fibers, resulting in improved dough rheology, enhanced flour functionality, and favourable nutritional attributes.^13^ In addition, its potential to reduce enzymatic activity and microbial contamination suggests a valuable role for ultrasound-assisted processing in extending shelf life and improving the safety of whole-wheat products.^20^

Traditionally, wheat grain proteins have been categorized into four Osborne fractions based on their solubility in water (albumins), saline solutions (globulins), aqueous alcohols (gliadins), or dilute acid or alkaline solutions (glutenins). Prolamins and glutelins-1 with superior water holding capacity (WHC), albumins and glutelins-2 (foaming capacity), and glutelins-1 and albumins (emulsifying capacity) are the Wheat Flour protein fractions with the most promise as functional additives in the food industry.^8^

Several recent reviews have examined ultrasound-assisted modifications of cereal-derived matrices. Wang *et al.* (2023)^21^ examined the effects of ultrasound treatment on cereal proteins, focusing on their functionality and structural characteristics across a broad range of cereal crops, without focusing on any specific crop. Zhang *et al.* (2023)^22^ reviewed the general applications of ultrasonication in flour-product processing, with greater emphasis on processing outcomes rather than protein-level structural changes. Vela *et al.* (2024)^23^ examined the physical modifications induced by ultrasonication in starches, grains, and flours, without specifically focusing on protein-related changes or linking these modifications to storage stability. In contrast, the present review focuses on how ultrasonication induces structural and chemical modifications in wheat proteins and how these changes affect their functional, processing, and storage properties within a unified wheat-focused framework. In addition, various analytical techniques used to characterize ultrasound-induced structural and functional changes are discussed, along with their effects on the quality and properties of wheat and wheat-based products.

## 2. Ultrasonication: mechanism and applications in food processing

Ultrasound is an emerging non-thermal food processing technique that enhances food quality while ensuring safety, particularly for products with heat-sensitive nutritional, sensory, and functional characteristics. It typically operates at low temperatures, though longer treatment durations may be required for effective microbial destruction and enzyme inactivation.^24^ The temperature may rise depending on the ultrasonic power and treatment time, highlighting the need for careful process optimization.

The principle behind low-power ultrasound involves the propagation of sound through food materials in the form of mechanical waves, creating alternating cycles of compression and decompression. Like any kind of waves, these waves are also specific in their characteristics, such as wavelength, velocity, frequency, pressure, and period. Their interaction with matter causes variations in the sound velocity and attenuation due to absorption and scattering mechanisms of the matter.^25^ Thus, the propagation of the sound waves through the material also depends on the type of matter present.

In ultrasonic treatments, acoustic energy is not directly absorbed by the molecules of the medium; instead, it is transformed through the phenomenon of cavitation. Sinusoidal ultrasound waves propagate through an aqueous medium, causing longitudinal displacement of particles. This results in rapid cyclical compression and rarefaction phases, leading to the formation of tiny, collapsing bubbles, a process termed cavitation. Bubble collapse releases a significant amount of energy, creating high pressures (up to 20 MPa) and localized temperatures as high as 5000 °C, which can induce both physical and chemical modifications in grains.^26^

The physical effects of cavitation include the generation of micro-jets, shear forces, shock waves, and acoustic streaming. Bubbles collapsing near solid surfaces produce asymmetrical micro-jets, shock waves, and rapid local increases in temperature and pressure (“hot spots”). The effective range of these micro-jets is approximately equal to the bubble diameter, which depends on the applied ultrasound frequency.^27^ Chemical modifications during ultrasonication arise from the formation of free radicals, such as hydroxide (˙OH) and hydrogen (˙H) radicals, resulting from the decomposition of water molecules within cavitation bubbles.^28^ These radicals can interact with food macromolecules, initiating oxidation–reduction reactions that modify starch, protein, and fiber structures and enhance nutrient bioavailability. Fig. 2 shows the mechanism of ultrasonic cavitation.

**Fig. 2:**
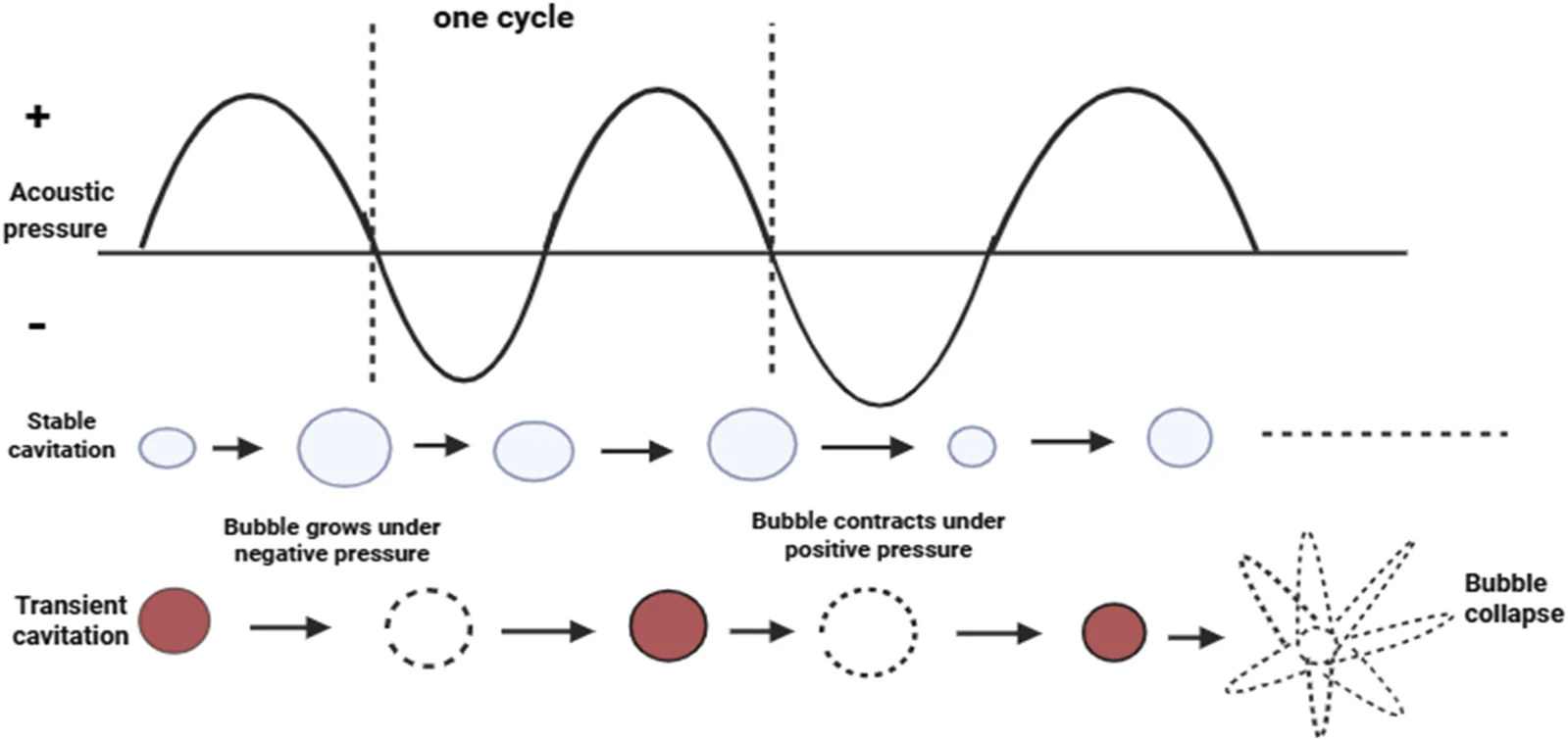
Mechanism of ultrasonic cavitation.

The effect of ultrasound depends on wave properties, including frequency, amplitude, power, and intensity. Frequency refers to the number of oscillations per second, while amplitude represents the maximum displacement from equilibrium. Cavitation bubble size is primarily influenced by ultrasound frequency and power. Acoustic power can be estimated calorimetrically using the adiabatic temperature change over time (d*T*/d*t*) during sonication:^29^

1 Acoustic power (AP) = *mC*_p_ (d*T*/d*t*)

where *m* is the mass, and *C*_p_ is the specific heat capacity.

Acoustic intensity (AI) is defined as the acoustic power per unit area (*A*):Acoustic Intensity (AI) = *P*/*A*

Acoustic power density (APD) represents acoustic power per unit volume (*V*):Acoustic power density (APD) = *P*/*V*

In addition, sonication duration, duty cycle, and intrinsic properties of the medium (pH, temperature, pressure, and structural characteristics) significantly influence ultrasonication efficiency and outcomes. Therefore, these parameters should be optimized as part of an integrated approach for maximal effect.^30^

Foods are complex matrices containing proteins, carbohydrates, enzymes, fats, vitamins, minerals, and water, requiring careful processing and preservation. Many food products, including wheat-based items, can harbour bacterial or viral contaminants, where heat treatment may be unsuitable due to potential nutrient and sensory degradation. This has necessitated the development of alternative non-thermal food processing technologies, such as high-pressure processing, cold plasma, pulsed electric fields, supercritical fluid extraction, ultraviolet irradiation, and ultrasound, to ensure microbial safety while preserving quality.^31^

Conventional thermal preservation methods, while effective in inactivating microbes and enzymes, can degrade heat-sensitive nutrients, alter flavors, and reduce sensory quality. Consumer demand for minimally processed foods that retain natural flavor, taste, and nutritional value has driven the development of non-thermal preservation techniques, including ultrasound. Ultrasound has proven to be a suitable technique for processing and preservation of food products due to its diverse applications in food systems, including extraction, drying, crystallization, filtration, defoaming, homogenization, meat tenderization, and preservation. Its ability to inactivate enzymes and microorganisms makes it suitable for food preservation.^32^

In food processing and preservation, ultrasonication has demonstrated advantageous results, such as increased product yields, shorter processing times, lower operating and maintenance costs, enhanced quality attributes, and reduced microbial contamination.^33^ In addition to improving food safety and quality, it provides chances to develop new products with special properties including extraction, yield, cloudiness, rheological characteristics, shelf life, and color, while reducing flavor loss, ensuring consistency, and permitting energy savings.^34,35^ Ultrasonication also conserves energy and enhances product quality by improving process efficiency and uniformity, thereby supporting more sustainable food processing^36^ (Table 1).

**Table 1:** Effect of ultrasonic treatment on wheat (grain) or wheat-based products

Source	Ultrasonic conditions	Findings	References
White-grain wheat (*Triticum aestivum* L.), a variety of Lubava	22 ± 1:65 kHz	Uniformly expanded grain structure and improved bread quality with reduced staling during storage	37
	252–630 W L^−1^		
Sponge cake	Bath type; 1 kW, 1.5 kW, or 2.5 kW; 3, 6, or 9 min; 27 °C	Improved cake texture and batter properties	38
Bread	Bath type; 1, 1.50, 2.05, and 2.50 kW,; 10, 20, 30, and 40 min	Improved the physical characteristics of bread and increased bread volume	39
Surimi-wheat dough and noodles	Bath type (20 kHz); 16.00, 18.67, 21.33, 24.00, and 26.67 W L^−1^, 30 min; 35 °C	Enhanced the dough network structure and improved the textural qualities of noodles	40
Gluten protein and noodle	Multifrequency:-single-frequency (40 kHz), dual-frequency (28/40 kHz), and triple-frequency (28/40/80 kHz) sonication; 67 W L^−1^; 10 min (pulsed time 10 s on/5 s off); 30 °C	Enhanced protein unfolding, thereby improving the functional characteristics of the product	41
Dough and steamed bread with 50% sweet potato pulp	Bath type (40 kHz); 7.5, 20.0, 22.5, 25.0, and 27.5 W L^−1^ 40 min; 32 °C	Enhanced the quality of both dough and steamed bread	42
Dough and gluten components	Probe type; 20, 40, 60, 80, or 100 W L^−1^; pulse (5 s on/5 s off); 20 kHz;−18 °C	Shortened the dough's freezing time and enhanced water migration	43
Bread	Bath type; 350 W; 40 min	Reduced the decline in bread quality and enhanced the longevity of bakery products	44
Gluten-free pan bread	Probe type; 0, 30, and 70% intensity	Reduced the firmness of bread and enhanced its sensory attributes	45
Noodles	Bath type; 66 W L^−1^; 5, 10, 15, 20, 25 and 30 min	Enhanced the quality of fresh wet noodles, reduced the firmness of steamed buns, and increased their shelf life	46
Dough	A probe operating at 20 kHz is paired with two flat plates set at 40 kHz; 35, 70, 105, 140, 175 W L^−1^; 2 to 10 min	Strengthened the mechanical properties of dough and enhanced the moisture retention of fermented dough	47
Steamed bread	Bath type; 0, 200, 300, 400, and 500 W; 32 °C; 40 min	Enhanced the structure of gluten protein	48
Wheat flour	Probe type; 600 W; 1 min	Enhanced flour quality and dough rheological characteristics	49
Wheat and sweet potato flour	Probe type; 20 kHz, 750 W; 2, 4, 8, 16, and 20 hours	Altered the functionality of flour and modified the structure of starch granules	50
Soy and wheat protein	Probe type; 20 kHz 95% amplitude; 34 W cm^−2^; 2 min	Improved the emulsifying properties and decreased the particle size of both proteins	51
Wheat gluten	Probe type; 540, 720, and 900 W; 10 min; 25 °C; 10.8, 14.4, and 18.0 W cm^−2^ intensity	Improved the emulsifying, foaming, and rheological properties of gluten samples	52
Gliadin in wheat and green wheat	Probe type; 0, 200, 300, 400, 500, 600 W; 30 min; 30 ± 2 °C	Improved the functional characteristics including solubility, emulsifying properties, water and oil retention	53
Wheat gluten with soy protein isolate	Bath type; 40 kHz, 300 W; 0, 10, 20, 30, or 40 min; <25 °C	Decreased particle size and enhanced the water-holding capacity and storage modulus	54
Buckwheat protein	Probe type; 60% amplitude; 5–30 min; pulse (10 s/5 s); 40–100% amplitude; 10 min; pulse (10 s/5 s)	Improved the digestibility of Buckwheat proteins	55
Dough sheets made from wheat and noodles	Bath type; 20 kHz, 360 W, ; 30–32 °C; 0–40 min	Enhanced the texture of noodles and shortened the resting period of dough	56

## 3. Effects of ultrasound on wheat grain composition and functionality

Cereal grains are widely consumed as the most significant source of carbohydrates, including dietary fiber, protein, and lipids. They represent a major source of energy for adult populations, providing approximately 30% of energy intake, 25–30% of proteins, 40–45% of carbohydrates, 40–60% of fiber, and substantial contributions of vitamins and minerals such as calcium (10–30%), selenium (20%), iron (40–45%), thiamine (25–35%), and folate (30–35%). Importantly, the processing method strongly influences the bioavailability of these nutrients; for example, fermentation can enhance nutrient bioavailability in grain-based products (Poutanen *et al.*, 2022).^57^ Rice, wheat, and maize are the three primary staples of the human diet, collectively providing over 35% of global caloric intake. Over the past six decades, production of all three has increased significantly, with maize production rising fivefold and rice and wheat tripling.^58^

Structurally, cereal grains are composed of two primary components: (i) the embryo, which develops into a new plant, and (ii) the starch-rich endosperm, which stores proteins and starch. The endosperm is composed of numerous cells containing proteins, carbohydrates, small amounts of lipids, and phenolic acids, all of which are responsible for providing nourishment to the developing embryo during germination.^59^

Whole grains are also rich in phytochemicals and other beneficial compounds, particularly in the bran and germ fractions.^60^ These fractions contain substantial amounts of micronutrients, dietary fiber, and phytochemicals and are often used as milling by-products or animal feed.^61–63^ Their reintegration into flour or use as functional food ingredients can improve the nutritional quality of human diets.^64^ Bran and germ fractions also provide indigestible fibers and substrates such as phenolic compounds, resistant starch, β-glucan, and arabinoxylans, which can act as prebiotics and influence gut microbiota activity.^65^

A complex mixture of proteins and starch, together with trace amounts of lipids and vitamins, makes up wheat dough, which is also used to produce flour-based products. The edible quality and processing characteristics of the final product are determined by the dough cohesion, strength, rheological properties, and ductility. Among these, the dough protein network structure plays a crucial role in determining its characteristics.^42^

The interaction between proteins and starch is essential for the formation of a strong dough structure. A continuous protein matrix forms the skeleton, which is filled with starch particles, creating a dense and stable structure^66^Fig. 3. Schematic representation of ultrasound treatment and its effects on the composition and structure of wheat grain. Similarly, the interaction between proteins and starch contributes to the formation of a solid dough structure, with the continuous protein matrix serving as a skeleton and starch particles filling the matrix to maintain a dense and stable structure.^67^ It was shown that the application of ultrasound to wheat flour dough reduces its surface hydrophobicity and reorganizes the secondary structures of gliadin, gluten, and glutenin. Additionally, ultrasound enhanced the structural characteristics of gluten.^31^

**Fig. 3:**
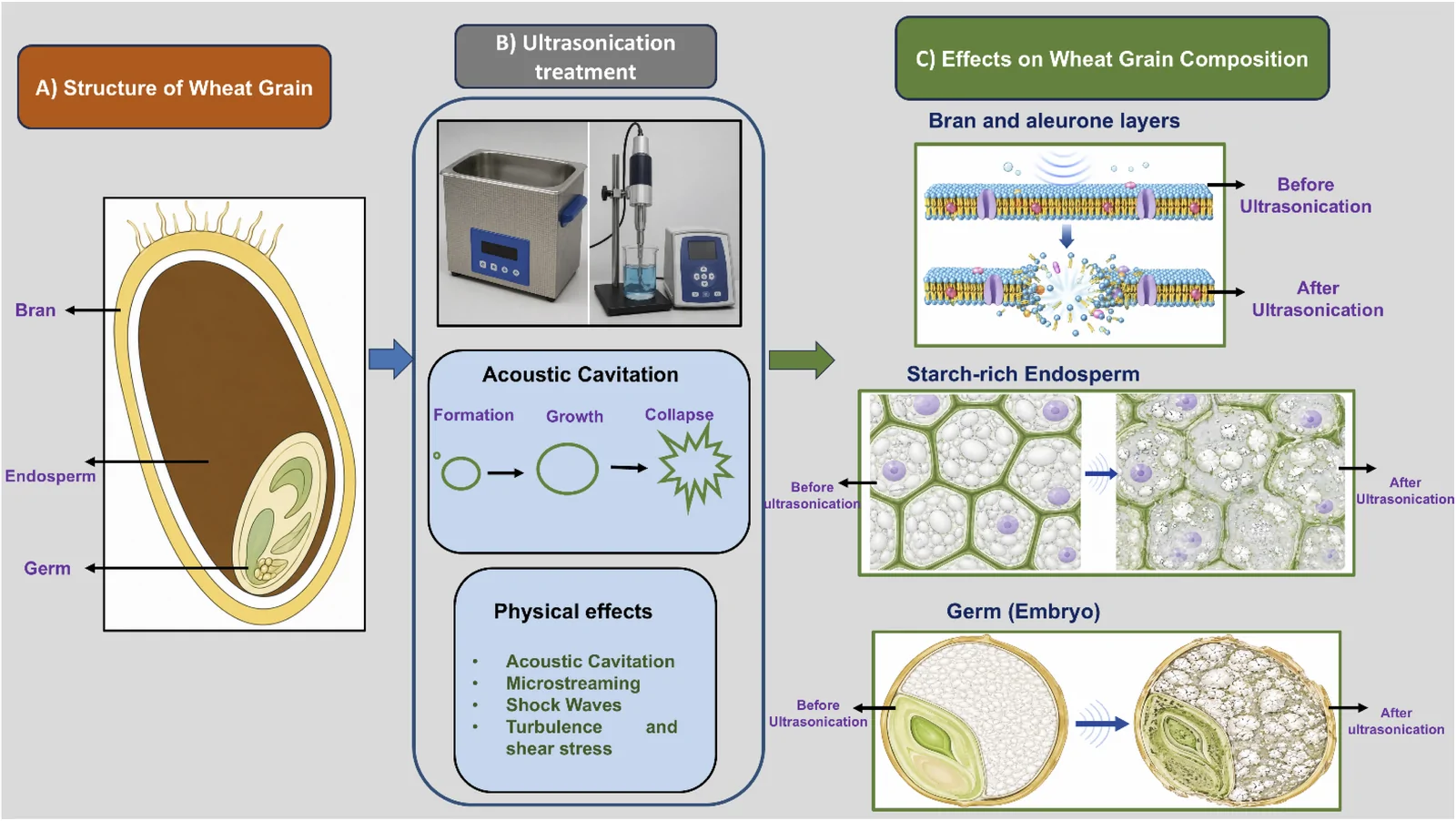
Schematic representation of ultrasound treatment and its effects on the composition and structure of wheat grain.

Habuš *et al.* (2021)^68^ reported that high-intensity ultrasound treatment of wheat bran (400 W, 80% amplitude, 15 min) decreased lipase and peroxidase activities by 63% and 90%, respectively, and increased the shelf life from 2 to 12 months. Similarly, Zhou *et al.* (2025)^69^ reported that wheat flour treated with single-frequency (60 kHz), dual-frequency (20 + 60 kHz), and triple-frequency (20 + 40 + 60 kHz) ultrasound showed enhanced ionic, hydrogen, and hydrophobic interactions following dual- and triple-frequency treatments at 24 and 72 h. These noncovalent interactions strengthen the gluten network by promoting interactions between protein chains and the solvent, as well as within proteins. Higher levels of hydrogen and hydrophobic interactions also promote protein polymerization and noncovalent binding between gelatinized starch and proteins.

### 3.1 Proteins: structural and functional modifications

Wheat proteins are classified into four categories: acid-soluble glutenins, alcohol-soluble prolamin (gliadins), salt-soluble globulins, and water-soluble albumins.^70^ A major wheat protein, gluten, is composed of glutenin and gliadin. Approximately 30–40% of total gluten proteins are glutenin, which has a fibrous, thin structure and a molecular weight of 1–30 × 10^5^ Da. It determines the elasticity of gluten due to its large surface area and strong molecular-binding capacity. Gliadin, with a molecular weight of 30–80 × 10^3^ Da, is a globular protein that accounts for 40–50% of total gluten proteins.^71^ These components combine to form a network structure with viscoelastic properties through disulfide, hydrophobic, and hydrogen bonds.^72^ Their combination forms a continuous gluten network during grain maturation, in which starch granules are embedded following the coalescence of the protein bodies.^73^

Other wheat proteins, such as globulins, are soluble in dilute salt solutions but insoluble in water, whereas albumins are soluble in both water and dilute salt solutions. Enzymes and enzyme inhibitors are among the regulatory, metabolic, and protective proteins that constitute the majority of the albumin/globulin fraction. Gliadins are insoluble in water and salt solutions but soluble in aqueous alcohols, such as 60% ethanol or 50% propanol.^74^

Protein structure can be altered by ultrasound treatment through acoustic cavitation, thereby enhancing protein functional properties.^75^ Ultrasonic exposure is an emerging nonthermal treatment technique that involves cavitation, dispersion, and thermal effects and can enhance the generation of physiologically active compounds, including primary and secondary metabolites, in plant-based foods.^76,77^ In food technology, sonochemical processes can produce several effects on the food matrix through the development and subsequent collapse of air bubbles resulting from high internal pressures of up to 2000 kPa.^78,79^ The cyclic compression and expansion generated by ultrasonic oscillation in a liquid system result in the formation of cavitation bubbles, which progressively grow over several cycles before collapsing. The collapse of these bubbles generates turbulence in the cavitation zone, shear energy waves, and extremely high temperatures and pressures.^42^

The cavitation phenomenon and microstreaming currents produced by ultrasound can generate high temperatures (5000 K) and pressures (1800 atm), thereby enhancing protein modification by altering their spatial structure. Ultrasound treatment can consequently produce beneficial effects on the structural and functional characteristics, as well as on the enzymolysis, of different proteins.^80^

The viscoelasticity of the dough initially increased and then decreased as ultrasound power increased. The stability of disulfide bonds increased with greater cross-linking among proteins, starch, and other components. Competition for water increased the gluten development time and delayed gluten formation. Water absorption by gluten proteins through hydrogen and disulfide bonds enhanced the viscoelastic properties of the dough and contributed to the formation of a stable gluten network.^81^

In wheat dough, these effects can be reflected in changes in the microstructure of both raw materials and the final product. The effects can occur when the whole food matrix is treated as well as when individual components, such as water, are subjected to ultrasonic exposure. Applying ultrasound during the water-treatment stage can improve the permeability of membranes in wheat grain shell cells, promote the development of the protein matrix, and enhance the expansion of starch granules in wheat dough.^37^

Ultrasound can also modify the surface characteristics of proteins, which are associated with their behavior at the interface between two different polar phases and primarily include foaming and emulsifying properties. Following ultrasound treatment, both foam capacity and stability can be enhanced because of the homogenizing effect of ultrasound, which promotes a more uniform dispersion of protein and fat particles.^82,83^ Ultrasonic treatment can increase the content of free sulfhydryl (–SH) groups by unfolding the tertiary structure of proteins and exposing previously buried sulfhydryl groups. In addition, acoustic cavitation can promote the cleavage of disulfide (S–S) bonds, resulting in the generation of additional free sulfhydryl groups.^84^

However, protein molecules in an aqueous medium may also undergo cross-linking because of the radicals and superoxide produced during the sonolysis of water. Furthermore, hydrogen peroxide generated during acoustic cavitation may oxidize free sulfhydryl groups to the undesirable sulfinic acid form.^85^ Consequently, because ultrasound influences protein structure, it can cause significant changes in protein functionality. Li *et al.* (2021)^86^ examined the effects of sonication on the functional properties of brewer grain protein using 250 W for 20 min at a 60% duty cycle. At 25 °C, the sonicated brewer spent grain protein showed higher fat absorption capacity (2.85 ± 0.06–3.10 ± 0.10 g g^−1^), emulsion activity index (38.69 ± 0.45–40.44 ± 1.68 m^2^ g^−1^), emulsion stability index (65.55 ± 1.01–84.40 ± 0.59%), foaming capacity (106.31 ± 1.19–126.92 ± 1.41%), and foam stability (84.78 ± 2.73–107.33 ± 2.20%) compared with the untreated sample extracted using the conventional method.

Excessive ultrasound power can cause overextension of proteins, while the decrease in absolute potential may be related to the exposure of polar sites. Excessive ultrasound treatment can also adversely affect the gluten network structure.^87^ The particle size decreased as the ultrasound treatment time increased, which may be attributed to ultrasound-induced structural expansion and aggregation.^88^ So, the effectiveness of sonication in modifying protein properties is influenced by several factors, including frequency, power or power density, intensity, treatment duration, temperature, and inherent protein characteristics, such as macromolecular structure.

#### 3.1.1 Surface hydrophobicity (H_0_)

Protein surface hydrophobicity (H_0_), a measure of the number of hydrophobic groups on the surface of a protein molecule in contact with the polar aqueous environment, is strongly correlated with functional characteristics.^89^ Ultrasonic waves can disrupt hydrogen bonds, electrostatic interactions, and hydration forces between protein molecules, thereby exposing previously buried hydrophobic groups and revealing more hydrophobic amino acid residues, which increases surface hydrophobicity with prolonged ultrasonic treatment.^90^

Yang *et al.* (2017)^91^ sonicated defatted wheat germ protein (DWGP) at 600 W, thereby exposing sulfhydryl groups on the protein surface and potentially increasing the free sulfhydryl (–SH) content. Strong ultrasonic cavitation generates high pressure and shear forces that can alter protein conformation, exposing previously buried sulfhydryl groups and hydrophobic regions to the surrounding medium. Similarly, Li *et al.* (2023)^92^ reported that sonication of wheat germ protein (WGP) at 400 W for 10 min increased protein solubility, surface-free sulfhydryl groups, and surface hydrophobicity, while decreasing surface charge and particle size. These structural changes contributed to the improved emulsifying properties of the protein.

Ultrasonication at 20/35 kHz induced fluctuations in SH and SS contents as treatment time increased. This indicates that the SH and SS concentrations in the wheat germ (WG) suspension were highly dynamic and influenced by mechanical stirring forces. Ultrasound treatment increased SH content.^93^ Similarly, the quantity of free sulfhydryl (–SH) groups in the soy protein isolate/wheat gluten mixture increased after ultrasound and microbial transglutaminase (MTGase) dual modification, promoting cross-linking.^21^ A similar trend was reported by K. Zhang *et al.* (2022)^53^ in a study on wheat gliadin (WG) and green wheat gliadin (GG) treated with ultrasound at powers of 0, 200, 300, 400, 500, and 600 W, which showed a significant (*p* < 0.05) increase in free sulfhydryl (–SH) groups and surface hydrophobicity, along with a reduction in particle size.

Additionally, cavitation processes can destroy protein molecules, reducing large aggregates into tiny fragments and enhancing the protein hydrophobic surface.^94^ Zhou *et al.* (2013)^95^ investigated the effect of ultrasonication at power intensities ranging from 63.7 to 573.25 W cm^−2^ on the surface hydrophobicity of defatted wheat germ protein (DWGP). The fluorescence peak intensity (420–540 nm) increased with increasing ultrasonic power intensity and reached its maximum when the protein was treated at 191.1 W cm^−2^. This may be due to the fact that sonication disrupts the hydrophobic interactions within protein molecules, leading to protein unfolding and the exposure of previously buried hydrophobic groups, which results in increased fluorescence intensity. At higher power intensities (318.5–573.25 W cm^−2^), the fluorescence intensity decreased, suggesting protein re-association and aggregation.

#### 3.1.2 Foaming properties

Protein foaming characteristics, structural flexibility, and surface hydrophobicity are interrelated. After sonication, cohesive viscoelastic films can trap air due to the quick diffusion of molecules at the air/liquid interface and molecular rearrangement, which can alter the foaming characteristics. Accordingly, foaming characteristics are closely related to other characteristics such as particle size, surface hydrophobicity, molecular weight, and structural flexibility.^96^

On wheat gluten, ultrasound treatment had a major impact on the dynamic rheological characteristics, protein emulsifying, and foaming of gluten samples. The samples treated at 900 W power level had the highest values and enhanced emulsifying capacity. Due to the partially unfolded wheat protein generated by the exposure of hydrophobic areas to ultrasound, the foam capacity and foam stability values of gluten samples treated with ultrasound steadily increased as power levels increased.^52^ Akharume & Adedeji (2023)^97^ showed that FC greatly improved for both the prolamin and glutelin fractions after a treatment using sonication (20 kHz; 12.70 mm probe; 100%, 75%, 50% amplitude; 5, 10 min). The Dawn prolamin FC increased from 3.83 to 10.33%, while the plateau glutelin FC increased from 22.50 to 34.33%. Furthermore, at 52.72 W for 5 min, FS increased from 57.50 to 100%. Other sonication parameters, like intensity (W cm^−2^) and power density (W mL^−1^), may also affect the protein foaming characteristics.

Nazari *et al.* (2018)^98^ investigated the effects of high-power ultrasound on millet protein concentrate (MPC) at intensities of 18.4, 29.58, and 73.95 W cm^−2^ for 5, 12.5, and 20 min. The results showed that foaming capacity decreased at low ultrasound intensity (82.37 ± 5.51 mL) but increased at high intensity (749.7 ± 2 mL). Similarly, Gharibzahedi and Smith (2020)^62,94^ reported that the foaming ability of soy protein isolate and faba bean protein isolate improved with increases in hydrophobicity, foam volume, and particle size, while pea protein isolate showed improved foaming properties up to 10 min of treatment.

#### 3.1.3 Emulsifying properties

The ability of proteins to emulsify mixtures is essential when using them as surfactants. Since proteins are amphiphilic, surface-active agents can create a solid, homogeneous emulsion of a protein in an oil-water mixture (Sun *et al.*, 2014)^99^ and enhance the emulsification characteristics, such as the emulsion stability index (ESI) and the emulsifying activity index (EAI). EAI measures the ability of a protein to generate emulsions, whereas ESI measures the capacity of a protein to create a stable emulsion over time.^86^

Zhang *et al.* (2022)^53^ reported that the functional qualities of wheat gliadin (WG) and green wheat gliadin (GG), such as their solubility, emulsification capabilities, ability to hold water and oil, thermal stability, and digestibility, were improved by ultrasound treatment. At 400 and 300 W, respectively, WG and GG had superior functional characteristics. According to these findings, ultrasonic treatment at the appropriate power was an effective technique for enhancing the functional properties of WG and GG. Furthermore, the protein size, conformation, surface hydrophobicity, and molecular flexibility under an ultrasonic field are all significantly impacted by the alteration of the Cereal Protein emulsification capacities.^96^

Hu *et al.* (2015)^89^ investigated the effects of high-intensity ultrasound (HUS; 20 kHz, 400 W) on soybean β-conglycinin (7S) and glycinin (11S) fractions for 5, 20, and 40 min. The results demonstrated that, with increasing treatment time up to 40 min, the emulsifying activity index (EAI) of the 7S fraction increased from 11.4 to 17.9 m^2^ g^−1^, while that of the 11S fraction increased from 11.2 to 14.2 m^2^ g^−1^. Similarly, Shokri *et al.* (2022)^100^ reported that ultrasonication of whey protein (WP) at 31 W cm^−2^ for 10 and 20 min and 69 W cm^−2^ for 10 and 20 min resulted in an increase in both emulsifying activity index (EAI) and emulsifying stability index (ESI).

#### 3.1.4 Sulfhydryl (SH) and disulfide bond (SS) content

Protein molecules are known to have essential functional groups called sulfhydryl (SH) and disulfide (SS). Their ratio can also affect how effectively proteins function, and both are crucial for preserving the stability of protein structures.^54^

The findings of Yang *et al.* (2017)^91^ on defatted wheat germ protein demonstrated that, in comparison to the control, the SH content improved significantly (*p* < 0.05) when the ultrasonic power intensity increased from 40 to 100 W L^−1^. The exposure of the sulfhydryl group at the protein molecule exterior surface may be the cause of the rise in SH. The high pressure and shear stress of the ultrasonic cavitation phenomena may cause the hidden sulfhydryl groups of proteins to become visible during the protein size reduction process. The content of disulfide bonds (SS) and free sulfhydryl groups (SH) in dietary proteins has a significant impact on their functional characteristics and is crucial for the development of rigid structures.

(J. Liu *et al.*, 2021)^101^ applied ultrasonication at a constant power of 200 W for different sonication times (15, 30, 45, and 60 min) and at a constant sonication time of 15 min with different sonication powers (130, 160, and 200 W). Disulfide bonds are formed between the sulfur atoms (–S–S–) through the oxidation of two sulfhydryl (–SH) groups. Conversely, the cleavage of disulfide bonds can generate sulfhydryl groups. Increasing the sonication time resulted in a significant (*p* < 0.05) increase in the SH content of the extracted proteins. After 60 min, the protein contained 107.56 µmol g^−1^ of SH, which was 31.31% higher than that of the control. Another study demonstrated that multifrequency ultrasound treatment could break disulfide bonds between protein molecules, resulting in the reduction of disulfide bonds to sulfhydryl groups and consequently increasing the sulfhydryl (SH) content, thereby altering the space of protein molecules.^102^

Moreover, another study (Qin *et al.*, 2016)^54^ applied ultrasonication at 40 kHz and 300 W to soy protein isolate (SPI) and wheat gluten. An increase in ultrasonic treatment time resulted in an increase in free SH content. Buried SH residues may become exposed due to the cavitation effect, while disulfide bonds can be broken by shear forces, turbulent flow, and high pressure.

At frequencies of 20, 28, 35, 40, and 50 kHz, ultrasound treatment of rice protein resulted in a decrease in SS content and an increase in SH content, which might be due to the oxidation of hydroxyl groups generated by sonication. Meanwhile, the hydrophobic groups buried inside the protein became exposed on the protein surface, resulting in decreased structural stability due to SS bond disruption.^103^

#### 3.1.5 Particle size

The size of protein particles significantly impacts their functionality. The ultrasound shearing and cavitation pressures led to large aggregates breaking into smaller aggregates. A reduction in particle size may result from using ultrasonic therapy for a longer period. However, extended durations of ultrasonic treatment (*e.g.*, 24 minutes) may also result in increased hydrophobic group exposure.^104^ X. Li *et al.* (2023)^92^ applied high-intensity ultrasonic treatment using different powers (200, 400, 600, 800 W) for 10 minutes or at different times (2, 4, 6, 8, 10, 15, 20 min) at 400 W to Wheat germ protein (WGP), together with low frequency (20 kHz). The analysis of protein conformation and molecular characteristics revealed that ultrasound processing improved solubility, and it also reduced WGP particle size and surface charge.

Another study reported a reduction in particle size when the ultrasonic power increased from 0 to 400 W. The particle sizes of wheat gliadin (WG) and green wheat gliadin (GG) decreased by 42.1% and 32.2%, respectively. This reduction was attributed to the cavitation effect, which breaks down large clusters into smaller fragments and disrupts the interactions between protein molecules.^53^ O'Sullivan *et al.* (2016)^51^ also reported a reduction in particle size in wheat protein following ultrasonication at ∼34 W cm^−2^ for 2 min.

Black bean protein isolate (BBPI) was ultrasonicated at a frequency of 20 kHz and powers of 150, 300, and 450 W for 12 and 24 min. An increase in particle size was observed, which might be due to the formation of soluble aggregates. Following low-power ultrasound treatment, turbulent forces and microstreaming may increase the rate of collision and aggregation, resulting in the formation of unstable aggregates and an increase in particle size (L. Jiang *et al.*, 2014).^105^ Ultrasound with appropriate frequency and power settings can reduce the particle size of Cereal Proteins (CPs). However, particle size may increase when a high ultrasonic power is applied.^106^

#### 3.1.6 Conformational structures

Ultrasonication has the ability to change the protein structural properties in addition to its functional ones. Protein structures are classified into primary, secondary, and tertiary structures based on the spatial arrangement of polypeptide chains. According to the types and conditions of ultrasonic therapy, the protein structure is impacted differently. Only the secondary and higher-order structures of the protein changed when the treated protein was subjected to ultrasonic cavitation, while the primary structure remained unchanged.^107^

H. Zhang *et al.* (2020)^43^ showed that multifrequency sonication altered the secondary and tertiary structures of wheat gluten protein (WGP), affecting its hydrophilic/hydrophobic characteristics and water absorption capacity. The α-helix, β-sheet, β-turn, and random coil structures were analysed using circular dichroism (CD). With increasing pretreatment duration or power, the α-helix content initially decreased and then increased, while the β-sheet and β-turn contents showed only minor variations compared with the control.^108^

Based on the previously mentioned research, while the secondary structure may be affected by factors such as the sonication device, intensity, duration, and frequency, a protein can experience alterations in its secondary structures and display a more relaxed and flexible conformation after undergoing ultrasonication caused by cavitation. It was evident that the dominant structure present in green wheat gliadin (GG) and wheat gliadin (WG) was the β-sheet, with WG displaying a significantly higher content of ordered structures (α-helix and β-sheet) compared to GG. Furthermore, after undergoing ultrasonic treatment, the α-helix content in both GG and WG was significantly diminished. In GG, the β-turn content also decreased, whereas the levels of β-sheet and random coils increased (*p* < 0.05). However, in WG, the amounts of β-sheet, β-turn, and random coil structures increased.^53^

Therefore, parameters related to ultrasonic operation, including ultrasonic power, duration, and temperature, may affect the primary, secondary, and tertiary structures of proteins.

### 3.2 Dietary fibers: structural alterations and functional impacts

Hipsley used the term “dietary fiber” in 1953 to refer to the indigestible parts of plant cell walls. The first definition of dietary fiber was proposed by Trowell and Burkitt.^109^ “Dietary fibers are the carbohydrates that are resistant to digestion and absorption in the human small intestine and ferment completely or partially in the large intestine”, according to a scientific definition given by the Association of Cereal Chemists (AACC) in 2001. Cellulose, lignin, hemicellulose, pectins, oligosaccharides, waxes, and gums are its constituents.^110^ According to Hippocrates, “wholemeal bread makes larger feces than refined bread”. In terms of its physiological advantages, including reducing constipation, this clarified the significance of fiber.^111^ Dietary fiber can be grouped into two categories based on its solubility: soluble dietary fiber (SDF) and insoluble dietary fiber (IDF). It is recognized that both types are connected to particular physiological and metabolic processes in humans.^112^

DFs have excellent interaction properties with water and oil. They can gel, swell, emulsify, absorb, and hold onto water and oil.^113^ Dietary fiber-containing cereals, like wheat, are an essential source of non-haem iron. However, other cereal components, like bran fractions, contain concurrent factors like phytate that decrease the absorption of calcium, zinc, and iron. Ultrasonication modification of corn bran resulted in the highest level of structural porosity, which ruptured the IDF cell walls and disrupted the linkages between cellulose and hemicellulose.^114^ Ultrasonic treatment also improved the structure of the Wheat Bran fibers.^71^ Dietary fibers not only improve gut health but also exhibit a wide range of biofunctional properties, including antibacterial, antioxidant, hematopoiesis-promoting, immunomodulatory, and anticancer effects.^115,116^ Food-based strategies for health maintenance, such as consuming whole grains, fruits, and vegetables high in dietary fiber (DF), can help prevent and treat long-term metabolic diseases such as obesity, type 2 diabetes, and hyperlipidemia.^117,118^

## 4. Ultrasound-assisted preservation of wheat: enzyme and microbial inactivation

Thermal processing is the most common and widely used method for inactivating microbes and enzymes in food products. However, these techniques can negatively affect the natural flavors and nutritional value of food products. As a result, researchers have been investigating new methods to meet consumer demand for higher-quality products with extended shelf life.^119^

### 4.1 Ultrasound-induced enzyme inactivation

Enzymatic reactions can lead to unwanted changes in many foods during processing and storage. While heat treatment is a common method for eliminating these enzymes, it often results in nutrient loss and can degrade food quality. For this reason, nonthermal technologies are being explored as an alternative to reduce enzymatic activity in foods. The sonophysical effects of ultrasound induce structural changes and generate microjets within the medium. These microjets exert enhanced shear forces on protein molecules, thereby affecting the catalytic activity of enzymes. Sonication-induced shear forces can cause the unfolding of protein secondary structures, exposing previously buried hydrophobic amino acid residues to the surrounding environment. However, the denaturation of enzyme protein structures generally requires longer sonication times and higher ultrasonic intensities.^30^

The various enzymes present in the bran and germ fractions of wheat kernels initiate numerous chemical changes that influence the compositional and functional properties of whole-wheat flour. Generally, wheat flour consists primarily of macronutrients, including starch, water, proteins, and non-starch polysaccharides, as well as lipids and ash. It also contains several technologically significant enzymes, primarily amylases, proteases, lipoxygenase (LOX), polyphenol oxidase (PPO), and peroxidase (POD). While these enzymes remain inactive during the storage of grain and flour, they become active when water is added. Carbohydrate enzymes such as amylases and cellulases break down starches and fibers. Proteases hydrolyse proteins into peptides and amino acids. Lipases and lipoxygenases act on fats and oils, releasing fatty acids and oxidation products. This activation plays a crucial role in determining the functional attributes of the flour and the grain.^120^

The short shelf life of whole wheat flour is primarily associated with lipid deterioration. During storage, lipids undergo enzymatic hydrolysis and oxidation, resulting in losses in flour functionality, palatability, and nutritional quality.^121^ The presence of bran and germ makes whole wheat flour more susceptible to lipolytic rancidity than refined flour. Lipase and esterase hydrolyze soluble and water-insoluble esters, producing free fatty acids (FFAs) that contribute to hydrolytic rancidity. In addition, PPO and POD can promote oxidative reactions, while LOX catalyzes the formation of hydroperoxides and peroxides from unsaturated fatty acids. These reactions contribute to oxidative rancidity and can also influence flour color and dough properties.^121,122^

Water content, metals, antioxidant activity, fatty acid composition, and storage temperature influence the rate of lipid oxidation in bran. Consequently, controlling lipid-degrading enzymes is an important strategy for improving bran shelf life. Habuš *et al.* (2021)^68^ investigated high-intensity ultrasound (400 W, 80% amplitude, 15 min), with and without cooling, for enzyme inactivation in wheat bran. Ultrasound treatment without cooling reduced lipase and peroxidase activities by 63% and 90%, respectively, and decreased antioxidant activity by 13%. The treated bran maintained an acceptable anisidine value for more than 12 months under ambient storage, compared with only two months for untreated bran. Thus, high-intensity ultrasound without cooling effectively improved the oxidative stability and storage life of wheat bran by reducing lipid-degrading enzyme activity. Cereal lipase is heat-labile at temperatures above 80 °C.^68^ At 30 and 50 °C, the PPO inactivation rates were 82.59% and 92.92%, respectively, after 120 min. At 60 °C, the inactivation rate was 96.25% after 30 min and reached 100% after 90 min. For POD, the inactivation rates at 30, 50, and 60 °C after 120 min were 6.59%, 49.92%, and 95.18%, respectively.^123^

### 4.2 Ultrasound-induced microbial inactivation

Microbial contamination of cereal grains immediately after harvest can occur from various sources, including dust, water, diseased plants, insects, soil, fertilizers, and animal waste. The bacterial families Pseudomonadaceae, Micrococcaceae, Lactobacillaceae, and Bacillaceae make up the majority of bacteria found in grains.^124^ Meanwhile, the primary molds present belong to the genera *Alternaria*, *Fusarium*, *Helminthosporium*, and *Cladosporium*, although other genera may also be present.^125^ The microbial composition of cereals is important for preserving grains and flours, as microorganisms can alter product quality at high moisture levels. For short-term storage, the maximum moisture content of wheat, corn, barley, and rice should not exceed 13%. Additionally, temperature and oxygen levels are important factors to consider during storage.^126^

Research on nonthermal techniques for microbial and enzyme inactivation has increased in response to consumer demand for fresh, high-quality, microbiologically safe, and stable foods.^127^ Compared with thermal processing, nonthermal methods generally operate at lower temperatures, thereby reducing undesirable changes in food quality. Ultrasound technology offers several benefits, including reduced treatment time, low energy consumption, and increased material throughput,^128,129^ making it a promising approach for food preservation. However, its effectiveness can be limited by the resistance of certain enzymes and bacterial spores. Therefore, ultrasound can be combined with temperature and/or pressure to enhance microbial and enzymatic inactivation.^36^

Ultrasonic activity causes bubbles to develop, vibrate, and collapse asymmetrically, generating microjets. Ultrasound can disrupt and stress microbial cell membranes, enhancing the uptake of antimicrobials and reducing microbial viability. Microbial inactivation occurs mainly through cell-wall disruption, intracellular amino acid oxidation, and DNA modification. However, its effectiveness depends on exposure duration, type of treatment, food matrix, and microorganism involved, as shown in Fig. 4.^20^ The cavitation effect generates mechanical forces that cause different types of physical damage to the cell wall. Microstreaming and shock waves promote mass transfer and cell-wall damage, while localized hot spots cause thermal damage. These locally high temperatures can compromise the integrity of the cellular layer.^130^

**Fig. 4:**
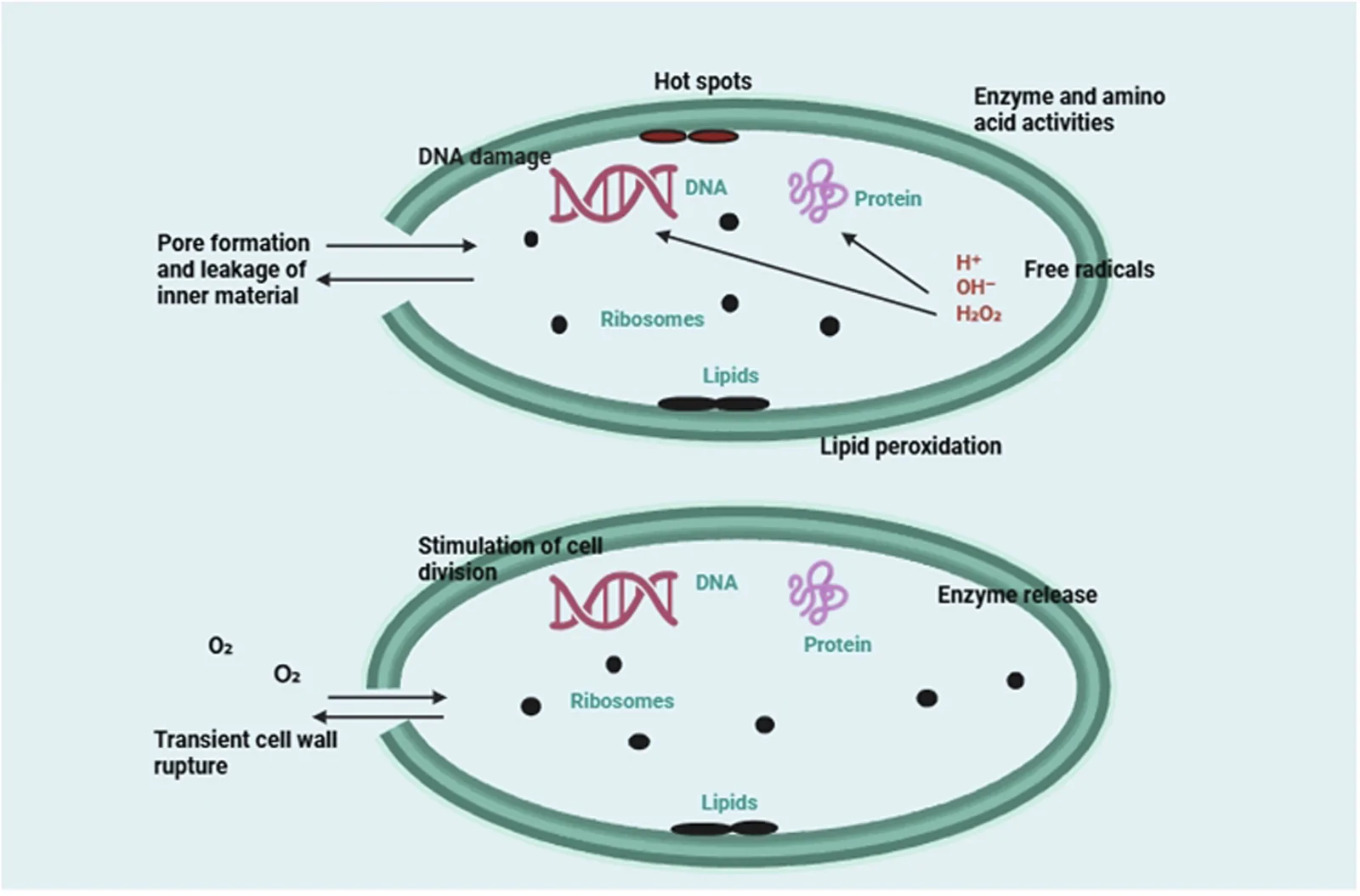
Mechanisms of ultrasound-induced microbial and enzymatic inactivation in grains.

The cell envelope of Gram-negative bacteria consists of an outer membrane containing lipopolysaccharides (LPS) and a thin peptidoglycan layer.^131^ In contrast, Gram-positive bacteria possess a thick peptidoglycan layer, typically 20–80 nm thick.^132^ The formation of intracellular ˙OH radicals and H_2_O_2_ induces the oxidation of intracellular amino acids, thereby inhibiting their specific functions.^67^ Chain reactions induced by free radicals result in lipid oxidation. These processes affect the fluidity, permeability, and integrity of bacterial membranes; ultimately, when free radicals enter the intracellular space, they damage internal components, leading to cell collapse.^133^ Schematic representation of microbial inactivation by ultrasound is shown in Fig. 5.

**Fig. 5:**
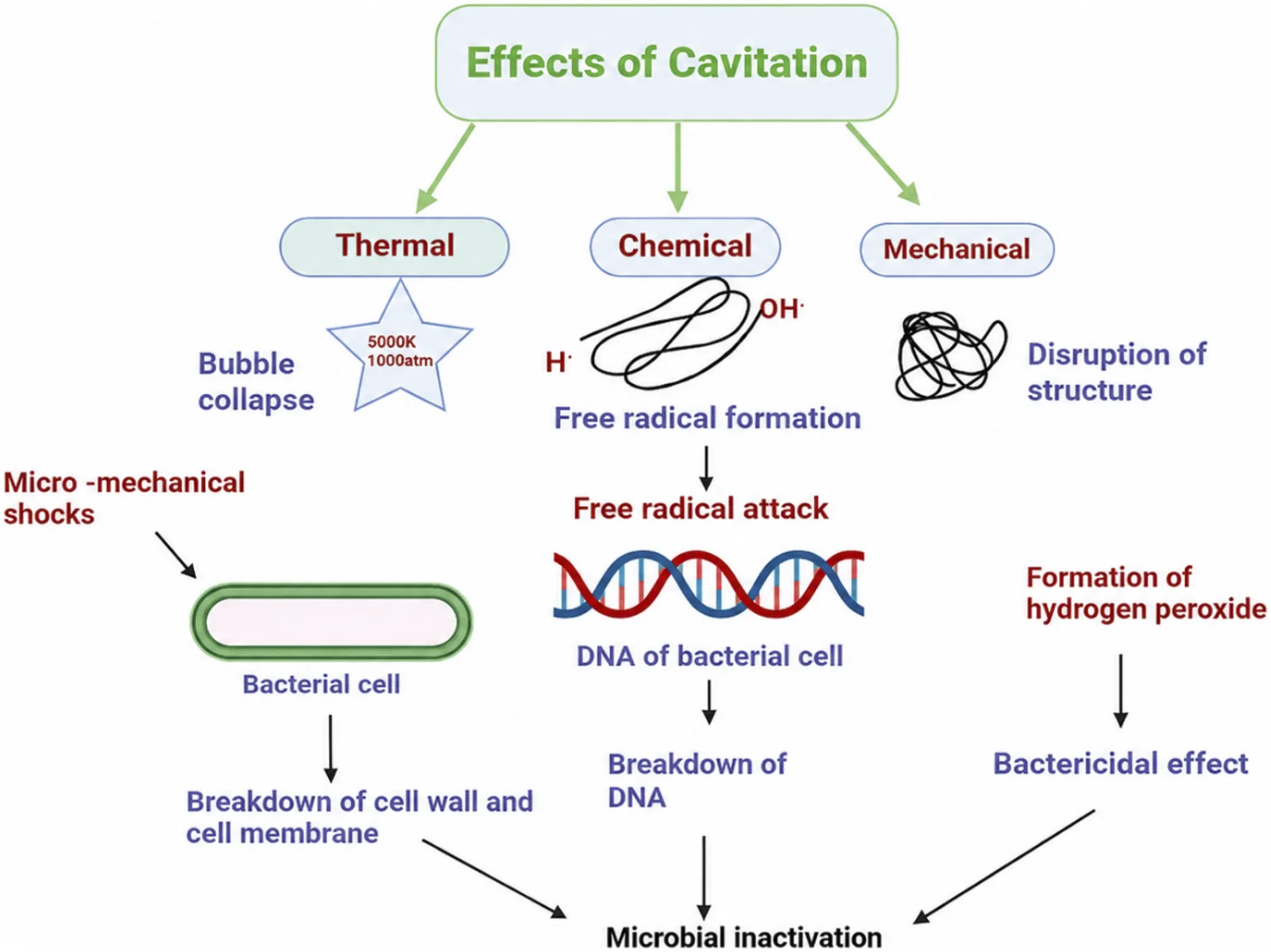
Schematic representation of microbial inactivation by ultrasound.

Bevilacqua *et al.* (2019)^134^ reported various microorganisms subjected to ultrasound treatment and suggested the following order of resistance: spores > fungi (molds) > yeasts > Gram-positive bacteria > Gram-negative bacteria. Thus, Gram-negative bacteria show the highest susceptibility, whereas bacterial spores show the highest resistance. Several factors, including cell size, shape, and external-layer morphology, influence this resistance.

Ultrasonic treatment, pulsed electric field, cold plasma, and ionizing radiation are four nonthermal techniques that mainly focus on improving the storage stability of grains.^135^ Cost-effective approaches for controlling microbial contamination in cereal crops included 13 physical, chemical, and microbiological decontamination treatments, which were evaluated using artificially contaminated grains before storage.^136^ Combining ultrasound with other nonthermal or biological approaches can enhance microbial elimination and inactivation.^137^ Spores are one of the major challenges in microbial inactivation because they are highly resistant to heat, radiation, oxidizing agents, and chemical treatments. Several nonthermal processing technologies have demonstrated the ability to inactivate both spores and vegetative cells, although their effectiveness depends on the microorganism and processing conditions.^138^ The microbial load of wheat is mainly present on the kernel surface; however, it can become distributed throughout the milled products during dry milling, resulting in poor microbiological quality of wheat flour. Although the low water activity of flour (*a*_w_ < 0.60) restricts microbial growth, contaminated spores and dormant microorganisms can persist for long periods, posing a potential food-safety risk, particularly in whole wheat flour.^139^

## 5. Analytical techniques for studying ultrasound-induced modifications

After applying any process, studying its effect on the commodity is necessary to determine the validity of the process. Scanning electron microscopy (SEM), Fourier transform infrared spectroscopy (FT-IR), rheology, and texture analysis are amongst the most commonly applied techniques for investigating ultrasound-induced modifications in wheat-based systems. These methods provide complementary insights, ranging from microstructural visualization to secondary structural changes and functional property evaluation.

### 5.1 Scanning electron microscopy

Scanning electron microscopy (SEM) is an effective tool for examining the surface characteristics of food materials at the micro- and nanoscale. SEM is widely used to analyze the surface topography and microstructure of various food materials, including fruits, vegetables, cereals, and processed foods. Surface morphology plays a crucial role in determining texture, moisture retention, and overall product quality during processing, storage, and consumption.^140^

Li *et al.* (2020)^46^ investigated the microstructure of noodle dough using scanning electron microscopy (SEM). In this study, a power density of 66 W L^−1^ was used, with ultrasonic treatment times ranging from 5 to 30 min. The SEM images revealed that the gluten proteins surrounding the starch granules appeared clumped, filamentous, and flaky, whereas the large and small spherical particles were identified as starch granules. Furthermore, the internal structure of the noodles was more compact, while porosity was observed in the outer layer. These structural characteristics may influence starch digestibility by contributing to both rapid and slow digestion during digestion.

Cao *et al.* (2023)^40^ revealed the microstructure of surimi-wheat dough, showing that gluten content in the wheat dough decreased as surimi was added. Consequently, the protein network structure was hindered, leading to a less uniform microstructure in the surimi-wheat dough. As ultrasonic treatment power increased, the beneficial effect on the dough gradually weakened, and large cracks appeared in the protein phase. This may be attributed to the disruption of disulfide (SS) and hydrogen bonds within the gluten network, resulting in reduced network integrity. However, both studies highlighted the role of the gluten protein matrix in dough microstructure. Li *et al.* (2020)^46^ reported that gluten proteins surrounded the starch granules, whereas Cao *et al.* (2023)^40^ observed that an increase in ultrasonic power resulted in the formation of large cracks in the protein phase.

Zhang *et al.* (2020)^141^ investigated the microstructure of wheat gluten protein (WGP)-enriched noodles using SEM. A dense and ordered structure was observed in the gluten protein without ultrasonication, whereas treatment with a dual-frequency mode (28/40 kHz) and a tri-frequency mode (28/40/80 kHz) increased the pore size from 10 µm without ultrasonication to 26 µm after sonication.

Similarly, in steamed bread, Song *et al.* (2022)^48^ reported that ultrasound induced protein conformational changes. SEM micrographs revealed that ultrasonication promoted the formation of a continuous and homogeneous gluten protein network, which was attributed to enhanced intermolecular interactions, particularly hydrogen bonding and van der Waals forces within the peptide chains. In agreement with these findings, Ulasevich *et al.* (2020)^142^ prepared gluten-free dumpling dough using ultrasonication at 35 kHz for 10–60 min. The dough contained smooth, rounded granules of gluten-free flour, with particle sizes ranging from approximately 75 to 525 µm as shown in the Fig. 6, and tiny particles accounting for more than 90% of the total particles. Although the dough structure was not initially fully homogenized, ultrasonic treatment produced a more uniform structure. Thus, although the two studies involved different types of dough, both reported that ultrasonication improved the uniformity of the dough structure. This suggests that ultrasound may improve the distribution and rearrangement of dough components. However, the extent and mechanism of these structural changes may depend on the composition of the dough and the presence or absence of gluten.

**Fig. 6:**
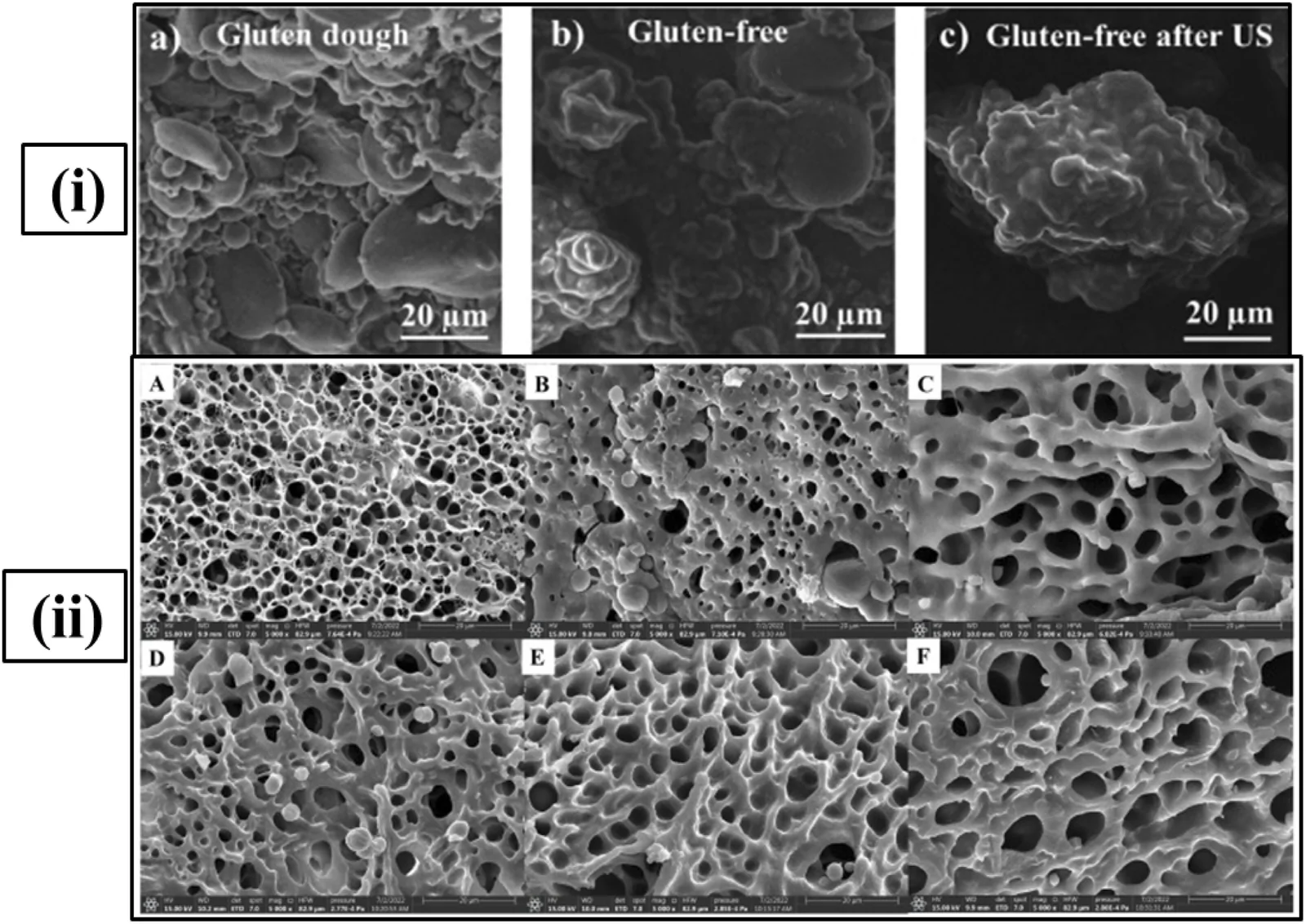
(i) The structure of the dough samples was analyzed using SEM images. (a) Gluten-containing dough, (b) gluten-free dough made without ultrasound treatment, and (c) gluten-free dough made with ultrasound assistance. (ii) The effect of ultrasonic power on gluten microstructure: (A) CSB (Chinese steamed bread); (B) control; (C) 200 W; (D) 300 W; (E) 400 W; (F) 500 W (reproduced from ref. 142 and 48 with permission from Elsevier, copyright 2023 and 2020).^48,142^

### 5.2 Confocal laser scanning microscopy

Confocal laser scanning microscopy (CLSM) is used to study the microstructure and microstructural evolution of the developing dough matrix, including gluten, starch, and other wheat flour components. During dough development, the structure evolves from unconnected flour particle aggregates composed of starch, fiber, and proteins into an interconnected protein network containing starch and other components. The developing network primarily consists of elastic polymeric glutenin structures, with gliadins embedded within them.^143^

Cao *et al.* (2023)^40^ examined the structure of the surimi-wheat protein network. The protein network exhibited a honeycomb-like structure. As ultrasonic power increased, the protein network became more uniformly distributed, less collapsed, and showed lower surface roughness. However, when ultrasonic power exceeded 21.33 W L^−1^, numerous holes appeared, and the protein network became loose and discontinuous. Lacunarity showed a reversed trend as ultrasonic power increased, whereas the protein percentage area, total number of junctions, and total chain length initially increased and then decreased. At an ultrasonic power of 21.33 W L^−1^, the cooked surimi-wheat noodles reached their maximum values for these parameters. Following ultrasonic treatment, the dough components could be more easily hydrated, which encouraged the development of a gluten network.

Similarly, Yao *et al.* (2021)^44^ investigated the microstructure of bread in which apricot kernel skin (AKS) partially replaced wheat flour to produce healthy bread. The gluten proteins are represented in red and yellow, whereas the starch granules are shown in green as shown in Fig. 7. The dark regions may correspond to pores, water, or other components that are not detected at the selected wavelength and magnification. The wheat dough showed a uniform, compact, continuous gluten network, with gluten proteins covering the starch granules. With the addition of AKS, gluten content decreased, resulting in a discontinuous, irregular gluten network with cracks observed within the gluten structure. After ultrasonic treatment, the starch in the AKS dough became slightly more evenly distributed. However, there were fewer large starch granules, and the starch polymerization degree decreased. This may be because ultrasound broke down and degraded the amylopectin structure, which increased the proportion of amylose and reduced starch aggregation.

**Fig. 7:**
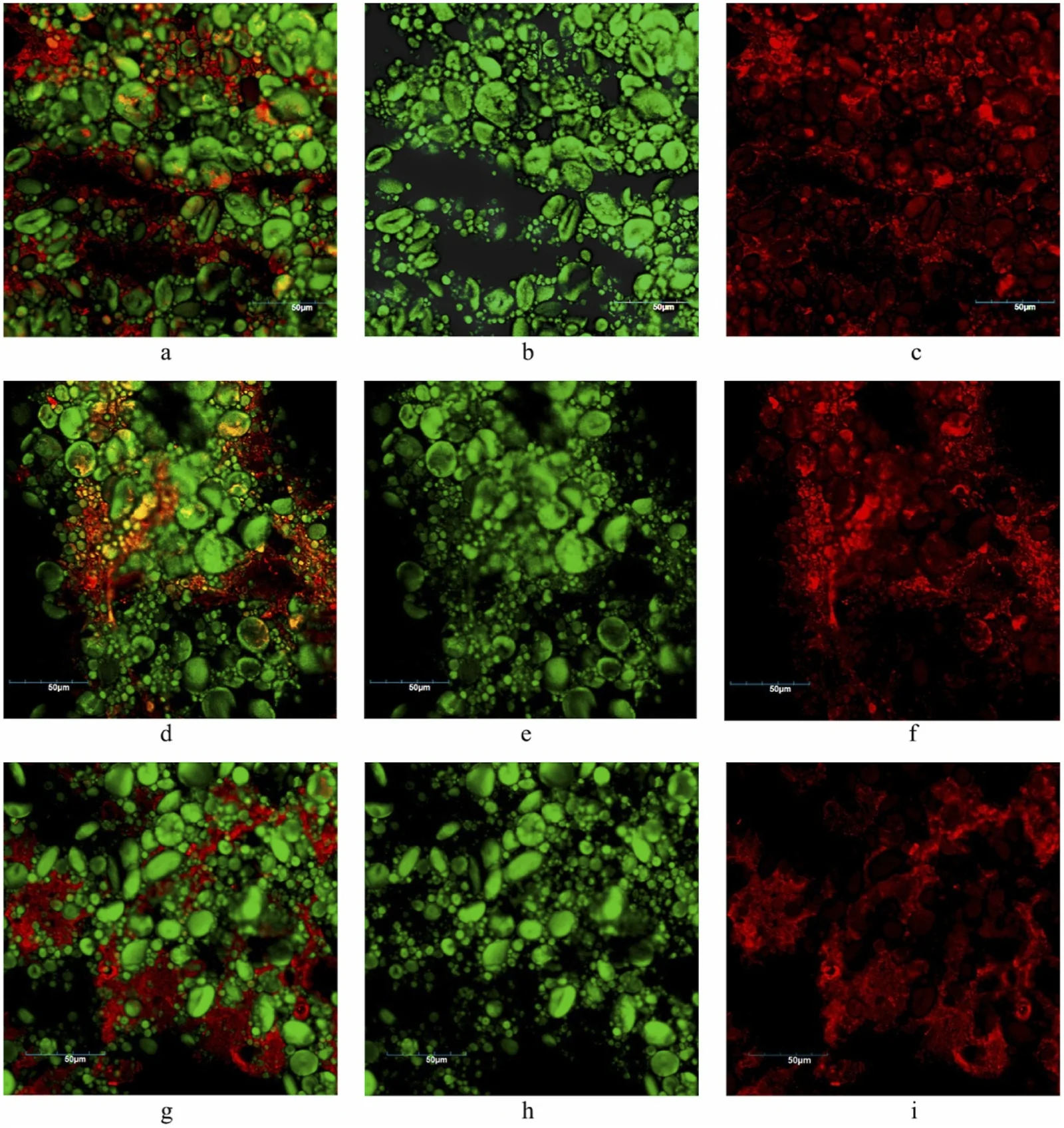
CLSM images of control dough (a–c), AKS-treated dough (d–f), ultrasonicated AKS-treated dough (g–i) (reproduced from ref. 44 with permission from Elsevier, copyright 2021).^44^

### 5.3 FT-IR

Fourier transform infrared (FT-IR) spectroscopy is a crucial tool for analysis and quality control in the food industry due to its simplicity and rapidity, as it requires no sample preparation. FT-IR absorbance bands can be used to identify the major components of biological materials, such as proteins, lipids, carbohydrates, and water, based on their functional groups and chemical linkages. The protein and starch composition of flour significantly influences dough quality.^144^

Li *et al.* (2021)^46^ performed FT-IR analysis of both raw and cooked flour and noodle samples to evaluate their protein characteristics. The RS100 mutant line exhibited a higher-intensity amide I band (1580–1720 cm^−1^) than the RS01 wild-type line. This finding is consistent with the observation that most heat-induced changes in gluten, as detected by FT-IR, were reversed upon cooling.

Li *et al.* (2019)^145^ reported improved wheat dough texture after ultrasound-assisted freezing. FT-IR analysis investigated the secondary structure of wheat gluten by analyzing the amide I region through deconvolution, second-derivative analysis, and Gaussian curve fitting. Ultrasound treatment had a significant effect on the secondary structure of wheat gluten. During the whole freezing process under ultrasound-assisted freezing, an irregular change in the α-helix content was observed with increasing ultrasonic power density; however, compared with the control, the α-helix content increased significantly at 80 W L^−1^. Under the maximum ice-crystal formation zone, the α-helix content initially increased and then decreased with increasing ultrasonic power density, reaching its maximum at 40 W L^−1^. These changes indicate that ultrasound treatment can modify the conformational organization of gluten proteins during freezing. The different regions of the amide I band were assigned to specific protein secondary structures: β-sheet (1615–1637 cm^−1^ and 1682–1695 cm^−1^), random coil (1637–1645 cm^−1^), α-helix (1645–1664 cm^−1^), and β-turn (1664–1682 cm^−1^).

Similarly, Zhang *et al.* (2021)^47^ investigated the effects of ultrasonic treatment on the dough fermentation and suggested an optimum treatment condition of 6 min at a power density of 105 W L^−1^. The amide I region in the FT-IR spectra of wheat gluten (WG) ranged from 1600 to 1700 cm^−1^. Ultrasonic treatment caused changes in the secondary structure of wheat gluten, with an increase in the proportions of β-sheet and random coil and a decrease in α-helix and β-turn. These changes suggest a transition from a more ordered to a relatively disordered protein structure, which may be attributed to the alteration of intermolecular forces and subsequent stretching or rearrangement of the protein structure during ultrasonic treatment. In addition to changes in the protein secondary structure, variations in the molecular structure of water were also observed following ultrasound treatment. The FT-IR spectrum exhibited a band around 3100–3400 cm^−1^, attributed to the stretching vibration of the –OH group and associated with hydrogen-bonding interactions among water molecules. When the ultrasonic power density exceeded 105 W L^−1^, the intensity of this band decreased, which was attributed to the disruption of hydrogen bonds between water molecules by ultrasound. Another study^146^ investigated the quality of dough using multifrequency ultrasound treatments for 10, 20, and 30 min, including single-frequency (20 kHz), dual-frequency (20/40 kHz), and triple-frequency (20/40/60 kHz) treatments as shown in Fig. 8. The spectral profiles for control, single-frequency, dual-frequency, and tri-frequency treatments from 10 to 30 minutes show similar patterns. Characteristic peaks appear at 1077.44, 1154.42, 1651.08, 2931.86 cm^−1^, and around 3291.12 cm^−1^. The absorption peak between 800 and 1200 cm^−1^ is due to the stretching vibrations of C–C, C–OH, and C–H bonds. The peak at 1651.08 cm^−1^ indicates C=O vibrations, while the peak at 2931.86 cm^−1^ indicates C–H stretching vibrations. The peak at 3291.12 cm^−1^ corresponds to O–H stretching vibrations, while the peak at 2348.97 cm^−1^ is caused by carbon dioxide stretching vibrations in the air. The absorbance ratio at 1077.44 cm^−1^ in the FT-IR spectra demonstrates the dough's short-range organized structure. Thus, studies demonstrated changes in the secondary structure of dough; however, the changes in individual structural components varied between the two studies. This may be due to differences in dough composition, moisture content, and ultrasonic treatment conditions.

**Fig. 8:**
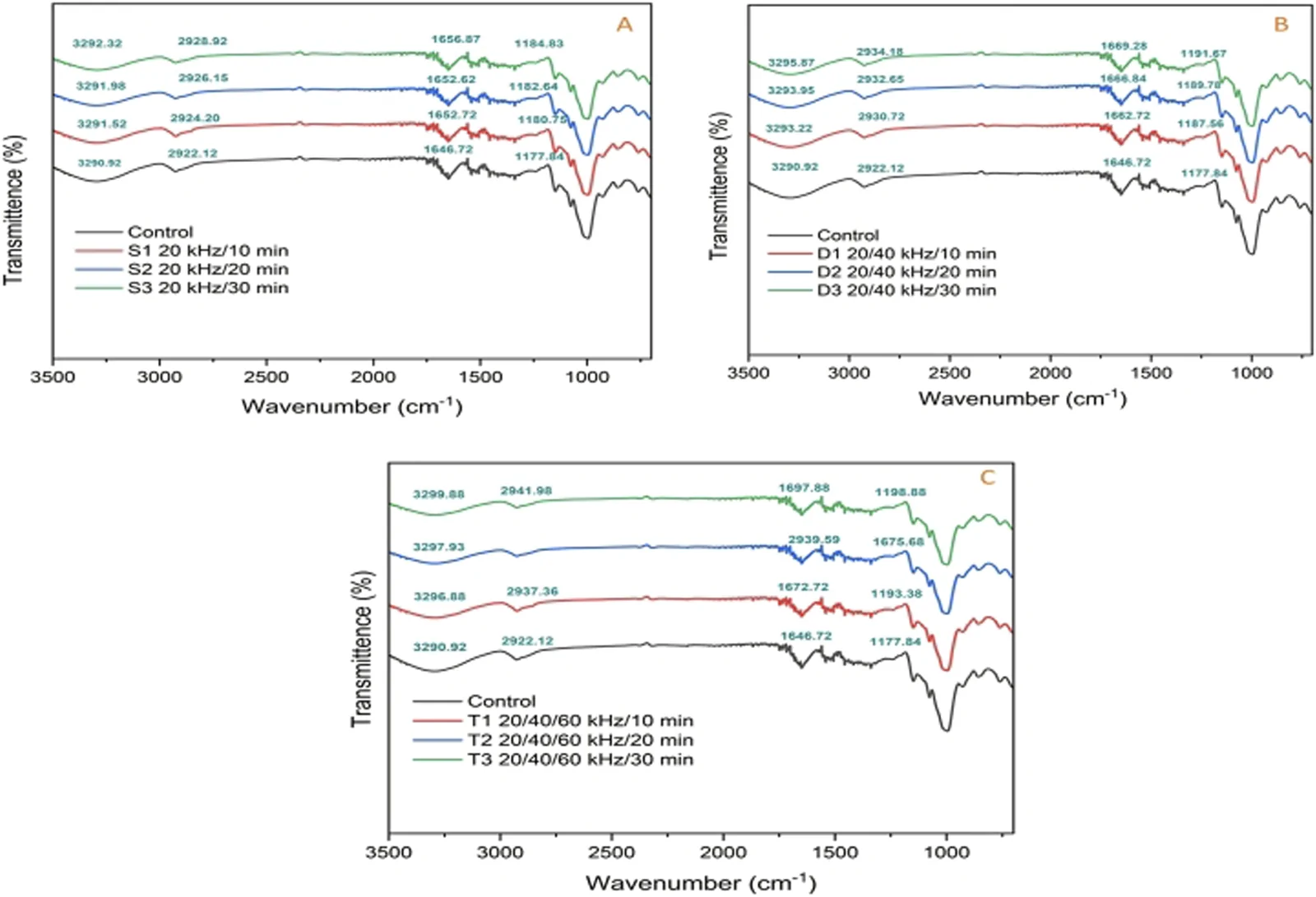
Fourier transform infrared spectra of frozen dough samples treated with different ultrasound frequencies and durations. (A) Control sample without sonication; S1: 20 kHz for 10 minutes; S2: 20 kHz for 20 minutes; S3: 20 kHz for 30 minutes. (B) Control sample without sonication; D1: 20/40 kHz for 10 minutes; D2: 20/40 kHz for 20 minutes; D3: 20/40 kHz for 30 minutes. (C) Control sample without sonication; T1: 20/40/60 kHz for 10 minutes; T2: 20/40/60 kHz for 20 minutes; T3: 20/40/60 kHz for 30 minutes (reproduced from ref. 146 with permission from Elsevier, copyright 2024).^146^

### 5.4 Rheological properties

Assessing the rheological characteristics of aerated batter is crucial for understanding its flow behavior and determining the requirements for producing high-quality products.^38^

*G*′ represents the elastic modulus (storage modulus), whereas *G*″ represents the loss modulus. Cao *et al.* (2023)^40^ reported that they used a variety of ultrasonic powers to treat dough that contains 50% surimi. The *G*′ was larger than *G*″ (*G*′ > *G*″) in 0–10 Hz for every dough sample, indicating that the elastic modulus predominated, as shown in Fig. 9. Both *G*′ and *G*″ decreased as the ultrasonic power increased from 21.33 W L^−1^, indicating a negative effect of higher ultrasound power on the dough structure. Strong ultrasonic forces may therefore disrupt polar hydrogen bonds and disulfide (SS) bonds between stable protein molecules, leading to the unfolding of wheat glutenin molecules and weakening the interactions between glutenin and gliadin. Ultrasound treatment produced an elastic dough with improved rheological properties that were suitable for dumpling molding.

**Fig. 9:**
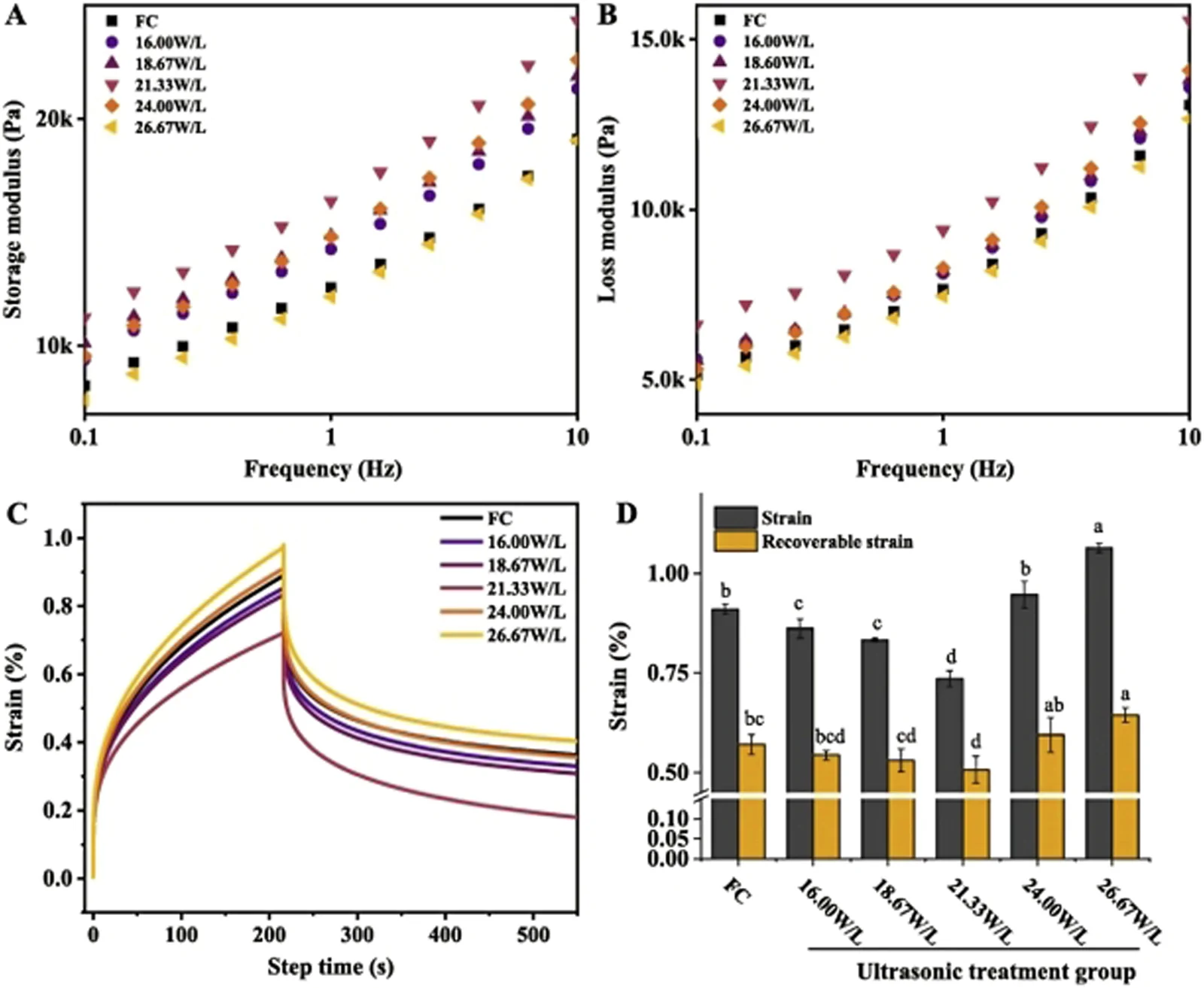
Dough's rheological properties were measured at 0.1–10 Hz (A): storage (*G*′) moduli, (B): loss (*G*″) moduli) and 0–550 s (C): strain, (D): recovery strain (reproduced from ref. 40 with permission from Elsevier, copyright 2023).^40^

The effect of multifrequency ultrasound treatment, including single-frequency (20 kHz), dual-frequency (20/40 kHz), and triple-frequency (20/40/60 kHz) treatments, on frozen dough was investigated. Compared with the control, the ultrasound-treated samples exhibited higher *G*′ and *G*″ values. The rise in *G*′ and *G*″ shows that gluten strength improves when proteins unfold during sonication. Acoustic cavitation causes these proteins to change shape and structure. As a result, starch granules spread more evenly through the dough, helping them absorb water and form a larger gel network. A strong, even gluten network is important for the dough's viscoelastic properties. The observed increase in gluten strength suggests that using ultrasound at certain frequencies and durations can help prevent large clumps from forming.^146^ Thus, the ultrasonic treatment improves the rheological properties.

### 5.5 Textural properties

Texture is an important feature in determining consumer preferences for food products. Hardness is a particularly significant parameter for evaluating the quality of wheat flour products, as it considerably affects the structural integrity of dough.^146^

In cake batter for sponge cakes, ultrasound was found to improve both the textural characteristics and cake volume.^38^ Sonication for nine minutes at different power levels consistently yielded greater sponge cake volume compared to the control, with a maximum 4% increase at 1 kW power. The cake hardness, springiness, gumminess, and chewiness were all significantly affected (*p* < 0.05) by ultrasonic parameters. Cakes made from batter treated at 2.5 kW for nine minutes displayed 12% lower hardness and 10% lower gumminess and chewiness than the control, while springiness, cohesiveness, and resilience were slightly improved. Similarly, Li *et al.* (2020)^46^ reported that ultrasound conditions affected the hardness and other textural parameters of fresh noodles. Fresh noodles treated at an ultrasound power density of 66 W L^−1^ showed a 33.43% increase in tensile force and a 14.19% decrease in hardness compared with the control group.

(Song *et al.*, 2023)^48^ Prepared steamed bread using ultrasound at a power of 400 W, which resulted in a higher specific volume than that of the control group. The addition of potato pulp (PP) degraded the gluten network and decreased the cross-linking of gluten proteins, which in turn reduced the cohesiveness and springiness of steamed bread with 30% potato pulp addition (SBPP). After 400 W ultrasonic treatment, the hardness of SBPP decreased by 5.28% compared to the control, while its cohesiveness and springiness increased by 2.60% and 6.90%, respectively, suggesting that ultrasonic treatment could improve the quality of steamed bread (Table 2).

**Table 2:** Effect of ultrasound on the wheat dough and wheat-based products

Food/material	Ultrasonic conditions	Hardness (g)	Spiringiness/elasticity	Cohesiveness	Gumminess (g)	Chewiness (g × mm)	Resilience	Reference
Surimi wheat noodles	16–26.67 W L^−1^	2322.37–3395.08	0.84–0.91	NR	NR	1346.98–1933.58	NR	40
Frozen wheat dough	20–60 kHz; 10–30 min; 45 W L^−1^	52.6–101.1	0.36–0.91	54.3–87.1	18.9–42.9	13.6–24.3	5.4–8.4	146
Steamed bread	200–500 W	2503.76–3078.77	0.88–0.93	0.78–0.80	1972.07–2423.61	1836.33–2124.27	0.39–0.44	48

The effectiveness of ultrasonic processing conditions, including power, frequency, and treatment duration, depends on the desired functional and structural properties of the dough. For example,^47^ study reported that ultrasonic treatment at 105 W L^−1^ for 6 min enhanced the structural and mechanical properties of the gluten network. Similarly, another study^141^ investigated single-frequency (40 kHz), dual-frequency (28/40 kHz), and triple-frequency (28/40/80 kHz) sonication. The water-holding capacity, oil-holding capacity, and solubility increased significantly (*p* < 0.05) with increasing ultrasonic frequency. Therefore, optimized conditions are essential. In contrast, increased ultrasonic power can disrupt the aggregate structure of gluten proteins, thereby reducing the wet gluten content. These findings provide evidence^100,147^ that an optimum treatment intensity is required, as both excessively low and excessively high ultrasonic intensities may adversely affect protein functionality. Consequently, it is essential to establish the optimal processing conditions for each type of food or food component. Ultrasound is a promising food-processing technique with a relatively low environmental impact and is therefore considered a green technology.^148^

Collectively, these findings demonstrate that SEM and CLSM provide detailed insights into microstructural modifications, FT-IR and related spectroscopic tools reveal secondary structure transitions, while rheology and texture analysis quantify viscoelastic and sensory-relevant properties. Together, they confirm that ultrasonication induces profound structural and functional transformations across diverse wheat-based systems.

## 6. Future scope and industrial applications

### 6.1 Current applications and limitations

The contemporary food industry faces growing responsibilities to deliver graded, nutritious, and minimally processed items, along with the standards of attributes desired by consumers. Such demands lead to a requirement for advanced sustainable processing technologies like ultrasonication, a green, non-thermal alternative to conventional processing.^149^ The effects of sonication on the structure of cereal proteins depend on the type of ultrasonic equipment used (probe or bath) and the conditions of the ultrasonic treatment (frequency, power density, and duration). The effects of ultrasound on the structure of cereal proteins, discussed in earlier sections, including reduced particle size, altered surface hydrophobicity, and modified secondary structure, may enhance properties like solubility, emulsification, and foaming capacity. Many such changes are highly desirable in industrial and processing operations. However, lack of standardized treatment parameters (frequency, power density, and duration) complicates cross-study comparison, and reaching generalized processing guidelines becomes difficult.

### 6.2 Industrial-scale feasibility and challenges

Translating the ultrasound technology from lab-scale to plant scale is a challenging affair.^150^ When processing different foods, it is important to carefully assess the distribution of pressure fields, temperature gradients, mass transfer rates, and liquid flow within the ultrasonic reactor. There are multiple limiting factors, the proper redressal of which is important for the translation of ultrasound technology at an industrial level. Foremost stands the scale-up. Laboratory systems deliver highly localized, high-intensity cavitation to small sample volumes, whereas industrial application requires continuous or multi-transducer systems capable of maintaining comparable treatment intensity across much larger throughput.^150^ Batch-type systems are straightforward but are not well suited to industrial-scale throughput. Continuous systems, although possible, have more complex engineering and less uniform acoustic energy distribution. Energy efficiency, an important factor determining process economics, presents another related concern.^151^ Ultrasound is usually considered a low-energy technology, but the power density needed to achieve measurable structural or microbial effects at industrial throughput has rarely been reported, which makes direct energy-efficiency comparisons with conventional processing difficult.^151^ The capital cost and cost per ton of this technology at industrial scale for wheat-specific applications has not been characterized.^149^ This lack of information makes it unclear whether the use of ultrasonication really justifies the shelf-life gain and functional property improvements of wheat. Lastly, the regulatory agencies are yet to come up with any guidelines regarding the use of ultrasound processing of grains, which is necessary for adoption of the technology at an industrial level.^152^ Considering all the research done on the use of ultrasound in food processing and the limitations listed above, it is clear that this technology is promising and well-validated at pilot scale, but not yet demonstrated at full commercial throughput for wheat-specific applications.

### 6.3 Research gaps and future opportunities

Although ultrasonication has been widely used to study its effects on wheat flour, dough, bran, and wheat-based products such as noodles, pasta, and bread, there remains a clear gap in research investigating the direct application of ultrasonication to whole wheat grains for shelf-life enhancement. Most of the existing research focuses on the modification of functional properties of proteins, starch, and fibers, as well as on improving the quality and texture of processed wheat products.

There are currently very few studies available on enzymatic and microbial inactivation in wheat grains with an aim to increase their shelf life. Habuš *et al.* (2021)^68^ reported that reducing the activity of enzymes involved in lipid breakdown could extend the limited shelf life of wheat bran. This study examined the effects of high-intensity ultrasound therapy (400 W, 80% amplitude, 15 min), with or without cooling, on the activity of wheat bran's lipase and peroxidase. When ultrasonic treatment was applied without cooling, 90% of peroxidase and 63% of lipase became inactive; nevertheless, the bran's antioxidant activity was also reduced by 13%. The ultrasound-treated bran was yellower, redder, and darker than the untreated bran. The anisidine value of unprocessed bran, when stored at room temperature, exceeded that after just two months, whereas the ultrasound-treated bran maintained a suitable anisidine value for over a year.

The quality of wheat is also influenced by the activity of the enzyme polyphenol oxidase (PPO), which oxidizes phenolic compounds and causes undesirable changes. Phenolic compounds are primarily found in the bran tissues of wheat. During milling, these compounds react with endogenous PPO, leading to color changes in wheat products. As a result, many wheat-breeding programs aim to develop varieties with relatively low levels of PPO in mature grains. The enzymatic browning of whole wheat flour, dough, and other products such as breads and noodles is caused by PPO. Additionally, browning in vegetables, fruits, and other cereal products is caused by polyphenol oxidases.^153^ In the food industry, heat treatment is typically employed to eliminate enzymes. Although PPO is quite susceptible to heat, POD shows higher resistance to it. As a result, fully inactivating POD through heat treatment may lead to a loss of heat-stable compounds and decrease the nutritional quality of the product. In wheat, PPO is found on the outer layer of the caryopsis, which is part of the bran. Interestingly, durum wheat has considerably lower PPO activity than hexaploid wheat variants.^154^

For microbial inactivation, Sarooei *et al.* (2018)^155^ reported that thermal techniques like pasteurization and sterilization are commonly employed to eradicate microorganisms; however, these methods can diminish nutritional content or negatively impact the texture, flavor, taste, and color. Hence, non-thermal treatments for microbial inactivation are preferred. This research evaluated the influence of both direct and indirect ultrasonic methods on the microbial load and chemical quality of raw wheat germ. The direct approach lowered the product's microbial count by 0.6 logarithms, whereas the indirect approach did not result in a significant reduction of the microbial load in the product.

Future studies should focus on the direct use of ultrasonication on wheat grains as an innovative preservation method. These investigations could assess the ability of ultrasound to deactivate enzymes that lead to lipid oxidation (such as lipases and peroxidases) and to diminish microbial contamination at the grain level, thus improving storage stability. Identifying the ideal sonication conditions for whole grains and combining this technique with other environmentally friendly preservation methods could offer a sustainable way to extend the shelf life of wheat while preserving its nutritional and functional properties.

Based on the research available and reported in this manuscript, the following specific research gaps are worth highlighting. There is a need to develop consistent sonication protocols with clear descriptions of process parameters such as frequency, amplitude, and duration, which will enable direct comparisons across studies and support eventual industrial guidelines. Studies focusing on the systematic evaluation of ultrasound effects on enzymatic and microbial inactivation at the whole-wheat-grain level, rather than at the fraction level (flour, dough, or bran), are unavailable. Pairing ultrasound with other mild preservation methods (*e.g.*, modified atmosphere, mild heat) to achieve additive or synergistic shelf-life extension (hurdle technology concept) without compromising nutritional quality.

The effect of ultrasound treatment on the sensory attributes of final treated wheat products, which is a direct indicator of consumer acceptance, remains sparse, despite the commercial importance. Techno-economic assessment of ultrasound-based technologies remains largely unexplored, limiting the evaluation of the feasibility of ultrasound against other established methods by the industry. As we look forward, it will be essential to refine ultrasound settings for various food categories, expand systems for large-scale production, and create uniform protocols to encourage widespread implementation. With ongoing research and improved accessibility, ultrasound is set to become increasingly important in promoting sustainable and efficient food processing. In the end, by combining our existing understanding and highlighting areas for innovation, we seek to offer direction to researchers, industry professionals, and technologists on how to implement ultrasound in their own consumer-focused, sustainable, and effective food processing projects.

## Conflicts of interest

There are no conflicts to declare.

## Data Availability

This review does not include any primary research findings, software, or code, and no new data were generated or analysed as part of this study.
